# Intention-to-treat analysis may be more conservative than per protocol analysis in antibiotic non-inferiority trials: a systematic review

**DOI:** 10.1186/s12874-021-01260-7

**Published:** 2021-04-19

**Authors:** Anthony D. Bai, Adam S. Komorowski, Carson K. L. Lo, Pranav Tandon, Xena X. Li, Vaibhav Mokashi, Anna Cvetkovic, Aidan Findlater, Laurel Liang, George Tomlinson, Mark Loeb, Dominik Mertz, for the McMaster Infectious Diseases Fellow Research Group

**Affiliations:** 1 Division of Infectious Diseases, McMaster University, Hamilton, ON Canada; 2 McMaster University Infectious Diseases Residency Program, JCC 3–71 at Juravinski Cancer Centre, 699 Concession St, Hamilton, ON L8V 5C2 Canada; 3 Division of Medical Microbiology, McMaster, University, Hamilton, ON Canada; 4 Global Health Office, McMaster University, Hamilton, ON Canada; 5 Leslie Dan Faculty of Pharmacy, University of Toronto, Toronto, ON Canada; 6 Department of Medicine, University Health Network and Mount Sinai Hospital, Toronto, ON Canada; 7 Institute of Health Policy Management and Evaluation, University of Toronto, Toronto, ON Canada

**Keywords:** Non-inferiority trials, Intention-to-treat, Per protocol, Systematic review

## Abstract

**Background:**

In non-inferiority trials, there is a concern that intention-to-treat (ITT) analysis, by including participants who did not receive the planned interventions, may bias towards making the treatment and control arms look similar and lead to mistaken claims of non-inferiority. In contrast, per protocol (PP) analysis is viewed as less likely to make this mistake and therefore preferable in non-inferiority trials. In a systematic review of antibiotic non-inferiority trials, we compared ITT and PP analyses to determine which analysis was more conservative.

**Methods:**

In a secondary analysis of a systematic review, we included non-inferiority trials that compared different antibiotic regimens, used absolute risk reduction (ARR) as the main outcome and reported both ITT and PP analyses. All estimates and confidence intervals (CIs) were oriented so that a negative ARR favored the control arm, and a positive ARR favored the treatment arm. We compared ITT to PP analyses results. The more conservative analysis between ITT and PP analyses was defined as the one having a more negative lower CI limit.

**Results:**

The analysis included 164 comparisons from 154 studies. In terms of the ARR, ITT analysis yielded the more conservative point estimate and lower CI limit in 83 (50.6%) and 92 (56.1%) comparisons respectively. The lower CI limits in ITT analysis favored the control arm more than in PP analysis (median of − 7.5% vs. -6.9%, *p* = 0.0402). CIs were slightly wider in ITT analyses than in PP analyses (median of 13.3% vs. 12.4%, *p* < 0.0001). The median success rate was 89% (interquartile range IQR 82 to 93%) in the PP population and 44% (IQR 23 to 60%) in the patients who were included in the ITT population but excluded from the PP population (*p* < 0.0001).

**Conclusions:**

Contrary to common belief, ITT analysis was more conservative than PP analysis in the majority of antibiotic non-inferiority trials. The lower treatment success rate in the ITT analysis led to a larger variance and wider CI, resulting in a more conservative lower CI limit. ITT analysis should be mandatory and considered as either the primary or co-primary analysis for non-inferiority trials.

**Trial registration:**

PROSPERO registration number CRD42020165040.

**Supplementary Information:**

The online version contains supplementary material available at 10.1186/s12874-021-01260-7.

## Background

In randomized controlled trials (RCTs), the most commonly analyzed populations are the intention-to-treat (ITT) and per protocol (PP) populations [1, 2]. The ITT population includes all patients, analyzed in their randomized treatment arms regardless of whether they took the treatment or completed the study [1]. In some studies, there are pre-defined modifications to the ITT population, such as including only patients who received at least one treatment dose [3]. This is sometimes referred to as modified ITT [3]. Hereafter, we use the term ITT population to include this modified ITT population. The PP population typically includes only patients who completed the study according to the protocol [1, 2].

ITT and PP analyses may differ in terms of how conservative the results are. Risk differences are usually calculated as success rate in the treatment arm minus the control arm, which is the absolute risk reduction (ARR). For the ARR point estimate and confidence interval (CI), the more conservative estimate would be smaller (more negative), which would favor the control arm more. Most non-inferiority trials use the lower CI limit to conclude on non-inferiority [4]. The treatment arm is non-inferior if the lower CI limit is bigger (more positive) than the non-inferiority margin. A more conservative and smaller (more negative) lower CI limit would be less likely to exclude the non-inferiority margin and thus more likely to reject non-inferiority.

ITT analysis is considered more conservative (less likely to find a difference between groups) than PP analysis in superiority RCTs, because the estimated treatment effect using ITT analysis may be diluted by inclusion of participants who did not receive the intervention [5]. In non-inferiority trials, however, this dilution and tendency towards making outcomes in the two treatment arms look similar may lead to inappropriate claims of non-inferiority [6–9]. Following this line of thought, PP analysis would be more conservative (less likely to declare non-inferiority) than ITT analysis and preferable as the primary analysis of non-inferiority trials [6].

Recent studies have challenged the notion that PP analysis is more conservative in non-inferiority trials. Simulation studies have identified scenarios where PP analysis was more conservative and other scenarios where it was not [10, 11]. However, there is little empirical evidence to date. One study did not find a significant difference between ITT and PP analyses in asthma trials [12]. Another study on antibiotic non-inferiority trials found a trend that ITT analysis may be more conservative than PP analysis, but was unable to draw definitive conclusions [13].

Of non-inferiority RCTs on drug therapy, anti-infective agents are the most common type of drug being evaluated [14]. For non-inferiority trials on antibiotics, the Food and Drug Administration (FDA) recommends ITT as the primary analysis [15–19] whereas the European Medicines Agency (EMA) recommends both ITT and PP as co-primary analyses [20]. We recently performed a systematic review on antibiotic non-inferiority trials [21]. In this secondary analysis, we compared ITT and PP analyses, with the aims of assessing (i) the claim that PP analysis is more conservative with respect to the point estimate as well as lower CI limit and (ii) whether the FDA or EMA recommendations should guide the preferred analysis and reporting strategies.

## Methods

This was a secondary analysis of a previously conducted systematic review (PROSPERO CRD42020165040) [21]. The review was conducted and reported according to the Preferred Reporting Items for Systematic Reviews and Meta-Analyses guidelines (checklist in Additional file 1: Appendix Text 1) [22].

### Data sources and selection criteria

We searched MEDLINE, Embase and the Cochrane Database of Systematic Reviews from inception to November 22, 2019. The detailed search strategy is described in Additional file 1: Appendix Text 2. We used the FDA drugs database to supplement our search [23]. For novel antibiotics that were approved by the FDA, we read through the drug approvals and labels to find the non-inferiority RCTs that supported the approval and were also published in journal articles.

We included studies published in English that were identified as non-inferiority RCTs in humans comparing two or more systemic antibiotic regimens used to treat a bacterial infection. Studies were included if the treatment and control arms were specific antibiotic regimens. Each arm within the trial should have a different antibiotic regimen.

Commentaries, reviews, study protocols, secondary analysis, and conference proceedings were excluded. We also excluded trial registrations where the results were not published in a journal article. Phase 2 and pilot studies were identified and excluded after full text reading.

To be included in this secondary analysis, the studies must have reported both ITT and PP analyses, and the outcomes in percentage absolute risk differences.

### Data extraction

Six reviewers screened abstracts after a training session to identify potentially relevant studies and extract full texts for reading. In the training session, all reviewers screened a sample batch of abstracts together and reached consensus on inclusion versus exclusion. The first 300 abstracts that each reviewer screened were double checked by another independent reviewer for consistency. If consistent, the reviewer then screened abstracts independently.

For full text review, two independent reviewers read and extracted the data in duplicate onto a standardized extraction form. Disagreements were resolved by discussion to reach consensus, and adjudication by a third reviewer if necessary.

### Variables collected

We extracted the following data from each journal article: journal, year of study, sample size, inclusion and exclusion criteria for ITT as well as PP population, treatment of missing data, and the primary outcome including the absolute numbers (successes and total number of patients in each arm) and reported CI.

### Primary outcome

**Fig. 1:**
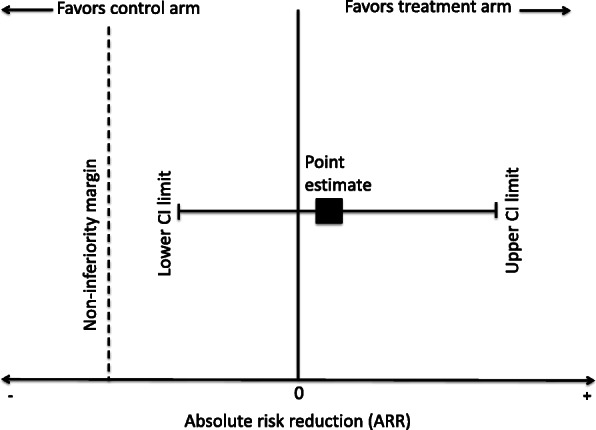
Orientation and interpretation of confidence interval relative to non-inferiority margin. CI = confidence interval

The co-primary outcomes were the point estimate and lower CI. We converted all risk differences to the standard ARR calculated as the success rate in treatment arm minus the success rate in the control arm, such that a negative ARR means that the results favor the control arm and a positive ARR means that the results favor the treatment arm. Based on this orientation, the lower CI limit can be interpreted as representing the worst plausible treatment effect for the treatment arm. A conclusion of non-inferiority was based on a comparison of this lower CI limit to the non-inferiority margin (Fig. 1).

We extracted the number of successes and total number of patients in the treatment and control arms to calculate the two-sided 95% CI for the ARR using the method described by Agresti and Caffo [24]. The Agresti-Caffo, Newcombe and Miettinen-Nurminen methods all perform equally well and are recommended as safe to use for sample size of 30 or greater [25]. We chose the Agresti-Caffo method, because it tends to have a more conservative CI width than the other two methods [25]. We also used the method described by Newcombe [26] to calculate the CI as a sensitivity analysis.

The more conservative approach between PP and ITT analyses was defined as the one with the smaller (more negative) lower CI limit, as the smaller limit is less likely to exclude a non-inferiority margin.

We used the calculated two-sided 95% CI to determine whether the treatment arm was non-inferior to the control arm based on the lower CI limit relative to the non-inferiority margin specified in the study. We then examined the concordance between the ITT and PP analyses. ITT and PP analyses would be concordant if both analyses reached the same conclusion. The analyses would be discordant if non-inferiority was proven in one analysis but inconclusive in the other analysis.

In the rare cases where a study that had two or more comparisons, we did not take into account the correlation of comparisons within studies.

### Risk of Bias assessment

Two independent reviewers assessed the risk of bias in duplicate based on the Cochrane Collaboration’s tool for assessing risk of bias in randomized trials [27]. Attrition bias was assessed for the ITT population.

The ITT and PP analyses were displayed on the funnel plot to assess for publication bias. Consider a scenario where non-inferiority was inconclusive in the ITT analysis and proven in the PP analysis. The authors may choose to omit the ITT analysis and publish only the PP analysis results. Therefore, it is possible that authors only report both ITT and PP analyses when both analyses successfully demonstrated non-inferiority. If this were the case, then there may be asymmetry in the funnel plot of ITT and PP analyses results.

### Statistical analysis

Descriptive analyses included number (percentage) for categorical variables and median (interquartile range IQR) for continuous variables. For comparison of point estimates, lower CI limits and CI widths between ITT and PP analyses in the same study, a paired Wilcoxon signed-rank test was used [13].

As an exploratory analysis, an univariate linear regression was used to estimate associations between study-level characteristics and the difference between the lower CI limit of the ITT and PP analyses. Possible predictors included the methods of dealing with missing data, risk for bias as well as inclusion and exclusion criteria for ITT and PP populations as binary variables. Variables with univariate *P* < 0.2 were entered into a multivariable linear regression model.

The excluded population is defined as patients in the ITT population who were excluded from the PP population. The total number of patients and treatment successes in each arm of the excluded population was calculated by subtraction, using the number of patients and treatment successes reported in each arm of the ITT and PP populations.

All tests were two sided with a *P* < 0.05 significance level. All analyses were done with R version 3.6.3 (R Foundation for Statistical Computing, Vienna, Austria). Funnel plots and Egger’s regression test for funnel plot asymmetry were done using the metafor package [28]. CI for ARR was calculated using the DescTools package [29].

## Results

### Studies included

**Fig. 2:**
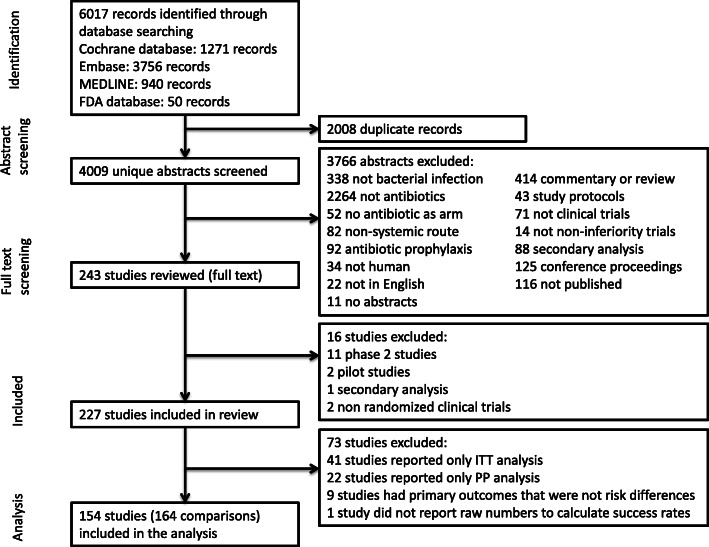
Flow diagram of study selection process

Of the 227 antibiotic non-inferiority trials, 41 (18.1%) studies reported only ITT analysis, 22 (9.7%) studies reported only PP analysis, and 164 (72.2%) studies reported both ITT and PP analyses. Furthermore, nine studies were excluded for reporting primary outcomes that were not proportions. One study was excluded because it did not report the numbers required to calculate the treatment success rates. Therefore, 154 (67.8%) studies met the inclusion criteria (Additional file 1: Appendix Table 1). Of these studies, eight studies had three arms and reported two comparisons. One study had four arms and reported three comparisons. Therefore, there were 164 comparisons included in the analysis (Fig. 2).

Of the 154 studies, 152 (98.7%) studies defined non-inferiority based on the lower CI limit with respect to the non-inferiority margin. Study characteristics with respect to the description and analysis of ITT and PP populations are described in Table 1.

**Table 1:** Study characteristics

	All comparisons within studies (*N* = 164)
Primary analysis population as per author	
ITT only	42 (25.6%)
PP only	45 (27.4%)
ITT and PP	55 (33.5%)
Not specified	22 (13.4%)
Sample size per group in the ITT population, Median (IQR)	221.5 (129.5, 326.0)
Proportion of treatment arm in the ITT population that was included in the PP population, Median (IQR)	0.86 (0.79, 0.93)
Proportion of control arm in the ITT population that was included in the PP population, Median (IQR)	0.87 (0.77, 0.92)
Definition of ITT	
Based on assignment alone	54 (32.9%)
Use of drug at least once	95 (57.9%)
Other exclusion criteria used	34 (20.7%)
PP population clearly defined	138 (84.2%)
Definition of PP population	
Exclusion based on concomitant therapy	96 (58.5%)
Exclusion based on incompliance	123 (75.0%)
Exclusion based on lost to follow-up	118 (72.0%)
Exclusion based on withdrawn from study due to other reasons	29 (17.7%)
Exclusion for other reasons	28 (17.1%)
Description of methods for handling missing data	64 (39.0%)
Missing data methods used	
Missing data as failure	58 (35.4%)
Tipping point analysis^a^	3 (1.8%)
Multiple imputation	4 (2.4%)
Last outcome carried forward	2 (1.2%)
CI reported	
2-sided 95% or 1-sided 97.5% CI^b^	142 (86.6%)

*CI* Confidence interval, *IQR* Interquartile range, *ITT* Intention-to-treat, *PP* Per-protocol

^a^Tipping point analysis assumes that all missing patients in the treatment group were failures and all missing patients in the control group were successes

^b^Other CIs include 1-sided 95% CI (*N* = 4), 2-sided 90% (*N* = 9), 2-sided 97.5% (*N* = 4). Five studies did not report any CI

### Risk of Bias

Risk of bias is summarized in Table 2. Risk of bias assessment for individual studies are described in Additional file 1: Appendix Table 2.

**Table 2:** Risk of bias assessment

	All comparisons within studies (*N* = 164)
Randomization	
High risk	3 (1.8%)
Low risk	110 (67.1%)
Unclear	51 (31.1%)
Allocation concealment	
High risk	3 (1.8%)
Low risk	74 (45.1%)
Unclear	87 (53.1%)
Performance bias	
High risk	75 (45.7%)
Low risk	84 (51.2%)
Unclear	5 (3.1%)
Detection bias	
High risk	58 (35.4%)
Low risk	100 (61.0%)
Unclear	6 (3.7%)
Attrition bias	
High risk	51 (31.1%)
Low risk	108 (65.9%)
Unclear	5 (3.1%)
Reporting bias	
High risk	28 (17.1%)
Low risk	136 (82.9%)
Unclear	0 (0%)

### Comparison between ITT and PP analysis

**Fig. 3:**
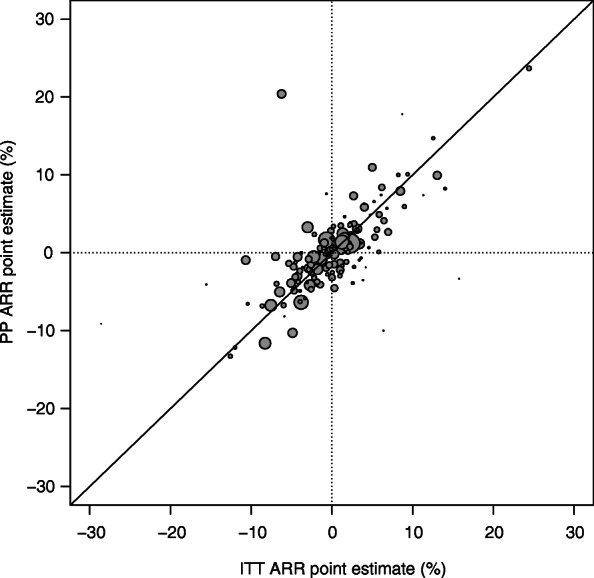
Graphical comparison of ITT versus PP point estimate. ARR = absolute risk reduction; ITT = intention-to-treat; PP = per protocol. The size of the points on the graph is proportional to the sample size of the ITT population. A diagonal line is drawn at y = x, so ITT analysis is more conservative for points above the line and PP analysis is more conservative for points below the line

**Fig. 4:**
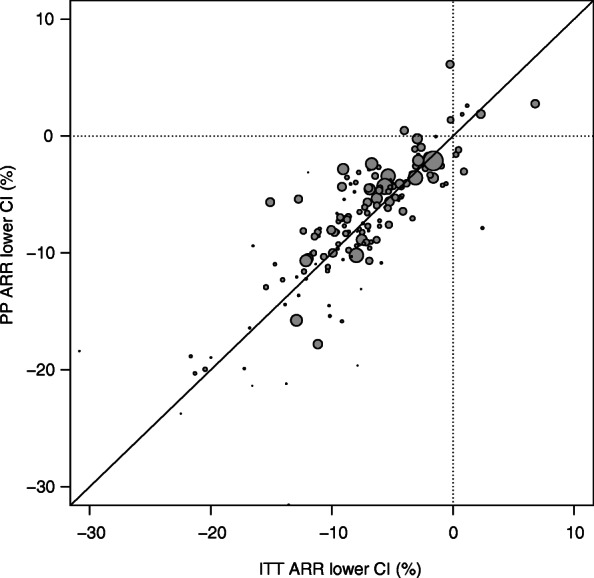
Graphical comparison of ITT versus PP lower CI limit. ARR = absolute risk reduction; CI = confidence interval; ITT = intention-to-treat; PP = per protocol. The size of the points on the graph is proportional to the sample size of the ITT population. A diagonal line is drawn at y = x, so ITT analysis is more conservative for points above the line and PP analysis is more conservative for points below the line. Three outliers were not included in this graph: 1) ITT lower CI of − 51.3% and PP lower CI of − 32.5%. 2) ITT lower CI of − 30.8% and PP lower CI of − 18.4%. 3) ITT lower CI of 15.7% and PP lower CI of 15.4%

Comparison of the results from the ITT and PP analyses are summarized in Table 3. Sensitivity analysis using the Newcombe method for calculation of CI yielded similar results (Additional file 1: Appendix Table 3). A forest plot for the ITT and PP analyses point estimates and CI is shown in Additional file 1: Appendix Fig. 1. The difference in point estimate and lower CI between ITT and PP analyses are shown in Additional file 1: Appendix Fig. 2. The point estimates from ITT and PP analyses were not statistically different (Fig. 3). Compared to PP analysis, ITT analysis had wider CIs (median of 13.3% vs. 12.4%; *p* < 0.0001) and more conservative lower CI limits (median of − 7.5% vs. -6.9%; *p* = 0.0402) (Fig. 4).

**Table 3:** Comparison of ITT to PP outcomes in terms of ARR

	PP Median (IQR)	ITT Median (IQR)	Difference ITT – PP Median (IQR)	Wilcoxon signed-rank test *p*-value	PP analysis is more conservative N (%)
Point estimate	−0.2 (−2.6, 2.2)	0.04 (− 2.6, 2.6)	− 0.01 (− 1.6, 1.9)	0.7025	81 (49.4%)
CI width	12.4 (9.7, 16.6)	13.3 (11.2, 17.5)	0.9 (−0.4, 2.0)	< 0.0001	58 (35.4%)
Lower CI limit	−6.9 (− 10.0, −4.0)	−7.5 (− 10.3, −4.7)	−0.5 (− 1.8, 1.2)	0.0402	72 (43.9%)

A positive value for the difference in CI width indicates less precise estimation of the ARR with ITT analysis. A negative difference for the lower CI limit signifies that the PP lower CI limit lies above the ITT CI limit, so ITT analysis has a more conservative result

*ARR* Absolute risk reduction, *CI* Confidence interval, *IQR* Interquartile range, *ITT* Intention-to-treat, *PP* Per-protocol

If the calculated two-sided 95% CI relative to the non-inferiority margin was used to determine non-inferiority, the results of the ITT and PP analyses would be concordant in 143 (87.2%) cases (Additional file 1: Appendix Table 4). Of the discordant cases, non-inferiority was proven in the ITT analysis but inconclusive in the PP analysis in 7 (4.3%) cases, whereas non-inferiority was proven in the PP analysis but inconclusive in the ITT analysis in 12 (7.3%) studies. Two comparisons did not provide a non-inferiority margin.

### Exploratory analyses

In both the univariate and multivariable linear regression models, the proportion of ITT population included in the PP population for the treatment group and control group had statistically significant correlations with the difference between ITT and PP lower CI limit (Tables 4 and 5). In the multivariable model, there was a trend where studies at low risk for allocation concealment bias and performance bias were associated with a smaller ITT lower CI limit. Multivariable linear regression weighted by the sample size in the ITT population yielded similar results (Additional file 1: Appendix Table 5).

**Table 4:** Univariate linear regression of difference between ITT lower CI and PP lower CI on study characteristics and risk for bias

Predictors	Co-efficient (95% CI)	*P*-value
ITT based on assignment alone	−0.21 (− 1.60 to 1.18)	0.7654
ITT based on use of drug at least once	0.01 (−1.31 to 1.34)	0.9823
PP exclusion based on concomitant therapy	−1.35 (− 2.66 to −0.04)	0.0439
PP exclusion based on incompliance	0.55 (−0.96 to 2.05)	0.4764
PP exclusion based on lost to follow-up	0.41 (−1.04 to 1.87)	0.5757
Proportion of treatment arm in the ITT population that was included in the PP population per every 10%	0.70 (0.09 to 1.32)	0.0247
Proportion of control arm in the ITT population that was included in the PP population per every 10%	−0.90 (−1.42 to −3.72)	0.0009
Missing data as failure	−0.68 (− 2.05 to 0.68)	0.3263
Tipping point analysis	− 2.66 (−7.53 to 2.21)	0.2818
Multiple imputation	−1.49 (−5.72 to 2.75)	0.4892
Low risk for allocation concealment bias	−0.87 (−2.17 to 0.44)	0.1936
Low risk for performance bias	−1.69 (−2.97 to −0.40)	0.0104
Low risk for detection bias	−1.21 (−2.54 to 0.11)	0.0728
Low risk for attrition bias	−0.56 (−1.93 to 0.82)	0.4264

The dependent variable in the model is ITT lower CI limit minus PP lower CI limit. Therefore, a negative co-efficient is associated with a smaller ITT lower CI limit, so the ITT analysis is more conservative than PP analysis. Conversely, a positive co-efficient is associated with a smaller PP lower CI limit, so the PP analysis is more conservative than the ITT analysis

*CI* confidence interval, *ITT* Intention-to-treat, *PP* Per-protocol

**Table 5:** Multivariable linear regression of difference between ITT lower CI and PP lower CI on study characteristics and risk for bias

Predictors	Co-efficient (95% CI)	*P*-value
PP exclusion based on concomitant therapy	−0.81 (− 1.93 to 0.31)	0.1558
Low risk for allocation concealment bias	−0.74 (− 1.82 to 0.35)	0.1810
Low risk for performance bias	−1.35 (− 2.94 to 0.24)	0.0960
Low risk for detection bias	0.70 (−0.97 to 2.37)	0.4076
Proportion of treatment arm in the ITT population that was included in the PP population per every 10%	2.89 (2.13 to 3.65)	< 0.0001
Proportion of control arm in the ITT population that was included in the PP population per every 10%	−2.73 (−3.37 to − 2.09)	< 0.0001

The dependent variable in the model is ITT lower CI limit minus PP lower CI limit. Therefore, a negative co-efficient is associated with a smaller ITT lower CI limit, so the ITT analysis is more conservative than PP analysis. Conversely, a positive co-efficient is associated with a smaller PP lower CI limit, so the PP analysis is more conservative than the ITT analysis

*CI* Confidence interval, *ITT* Intention-to-treat, *PP* Per-protocol

The median estimated ARR was 0% (IQR − 5.9 to 3.2%) for the excluded population and − 0.2% (IQR − 2.6 to 2.2%) for the PP population (*p* = 0.4335) (Additional file 1: Appendix Figure 3). The median success rate for the treatment and control arms combined was 44% (IQR 23 to 60%) in the excluded population and 89% (IQR 82 to 93%) in the PP population (*p* < 0.0001) (Additional file 1: Appendix Figure 4). The success rate for the treatment arm in the excluded and PP population are shown in Additional file 1: Appendix Figure 5, whereas the success rate for the control arm in the excluded and PP population are shown in Additional file 1: Appendix Figure 6.

The Egger’s regression test for funnel plot asymmetry of all ITT and PP analyses (Additional file 1: Appendix Figure 7) had a *p*-value of 0.9132. The funnel plots for ITT analyses only and PP analyses only are shown in Additional file 1: Appendix Figure 8 and 9 respectively.

## Discussion

In this systematic review of antibiotic non-inferiority trials, ITT analysis was more conservative than PP analysis in the majority of cases. In general, ITT analysis had wider CIs and more conservative lower CI limits than PP analysis. Although the difference between the lower CI limits of the ITT and PP analyses were small on average, there was a substantial variation at the individual trial level. For example, in two studies, this difference was larger than the non-inferiority margin itself. The substantial variation at the individual study level led to different conclusions on non-inferiority by ITT and PP analyses in approximately 12% of studies if non-inferiority was determined based on our calculated two-sided 95% CI relative to the specified non-inferiority margin in the study.

Although one might expect that the larger sample size in ITT would result in a narrower CI, the opposite was true in our study. The success rate of the excluded population was on average half that in the PP population in both the treatment and control arms, as shown in Additional file 1: Appendix Figs. 4,5 and 6. There are two ways that could lead to lower success rate in the excluded population. First, failure could occur more often in patients who could not adhere to treatment protocols or complete the study. Second, counting missing data as failure was the most common method of handling missing data and would significantly lower the success rate of the excluded population. As a result, the ITT analysis, which uses the combined PP and excluded population, tends to have an overall success rate closer to 50%, the value that maximizes the variance of the estimated ARR, resulting in a larger variance and thus a wider CI in the ITT analysis [13]. Since ITT and PP analyses had on average similar estimated ARRs, the wider CI was the reason for the ITT analysis being more conservative. In a trial with a success rate in the PP population that was 50% or lower, if the excluded population had a still lower success rate, then the net effect would be a narrower CI in the ITT analysis than in the PP analysis. This hypothetical example supports our finding that it is not possible to make a simple universal statement about the relative conservatism of ITT and PP analyses.

From a study design perspective, ITT and PP analyses measure two different treatment effects. ITT analysis measures the effect based on allocated intervention. In contrast, PP analysis measures the treatment effect of patients who started, adhered to and completed follow-up. From this perspective, it is expected that the treatment effect from the ITT analysis would have a lower success rate and be more conservative.

The multivariable linear regression model showed two noteworthy correlations. A more conservative ITT lower CI limit was associated with a lower proportion of the ITT population included in the PP population for the treatment arm and a higher proportion of the ITT population in the PP population for the control arm. These variables determine the proportion of the excluded population, which would then affect the CI width as described above. The linear regression model was only an exploratory analysis for the following reasons. First, for predictors used in the model, the methods were frequently not described in detail in the journal articles. For example, only 39% of studies described how they handled missing data. Second, many other factors may have contributed to which analysis would be more conservative such as pattern of missingness and non-compliance [11]. Data can be missing at random or missing in relation to treatment response [10, 11]. Non-compliance can also be related to treatment response, or study arm if there were differences in adverse effects [10]. These factors cannot be captured from empirical evidence. Lastly, the exclusion criteria for ITT and PP analyses were heterogeneous across studies.

Prior to our study, only two studies have compared ITT and PP analyses. These two studies included 11 and 20 trials, respectively [12, 13], whereas our study included 154 trials. Ebbutt and Frith found wider CIs in PP analysis and otherwise no consistent pattern of differences in either direction between the two analyses [12]. In contrast, maybe due to the larger number of trials in our systematic review, we found that ITT analysis had wider CIs and tended to be more conservative, a finding that is consistent with the study by Brittain and Lin [13].

Our study raises questions about whether ITT or PP analysis is more conservative in non-inferiority trials. While PP analysis may be more conservative than ITT analysis in theory, the empirical evidence here suggests that ITT analysis can be more conservative than PP analysis in practice. The difference in results between the two analysis strategies will depend on many factors and as a result, there is no justification for the omission of ITT analysis in non-inferiority trials. The PP population excludes patients based on post-randomization information such as missingness and compliance, introducing the potential for bias [10]. These considerations suggest that ITT should be the primary or co-primary analysis in non-inferiority trial of antibiotics, in line with the current FDA and EMA recommendations for reporting of non-inferiority trials [15–20]. There is room for improvement in reporting of ITT analysis in non-inferiority trials. For example, in our systematic review, approximately 10% of non-inferiority trials did not report an ITT analysis and 27% of non-inferiority trials that reported both ITT and PP analyses used PP analysis as the primary analysis.

Since the success rate of the ITT population that was excluded from the PP population significantly impacts the CI for the ITT analysis, the handling of missing data in ITT analysis has important consequences on conservatism. Future non-inferiority trials should pay attention to the methodology of how to handle missing data and describe it in detail in the publication. In our study, only 39% studies described how missing data was handled. Of the ways to handle and impute missing data, counting missing data as failure is the most common method. This would decrease the success rate in the ITT population and likely lead to a wider and more conservative CI. From the perspective of conservatism, this is likely an appropriate method in most studies. It should be noted that the tipping point analysis where missing data were counted as failures in the treatment arm and successes in the control arm has been used in trials and likely yields an even more conservative result.

The strength of our study is in the systematic and comprehensive literature search that includes the largest number of non-inferiority trials to date for comparison of ITT and PP analyses.

The study has several limitations. First, most abstracts were screened by a single person. However, the first 300 abstracts screened by each reviewer were doubled checked by another person to ensure consistency in the screening process. Second, there may be publication bias. We were only able to analyze studies that reported both ITT and PP analyses. For studies that reported either ITT or PP analysis only, it may be possible that the other analysis was omitted on purpose because it was too conservative and resulted in the study being a negative study. However, the funnel plots (Additional file 1: Appendix Figs. 7,8 and 9) and Egger’s regression test did not reveal any significant asymmetry. Third, our study described non-inferiority trials on antibiotics. Non-antibiotic trials may be different. For example, the proportion excluded from PP analysis based on compliance would be much higher for a trial on an oral cardiac medication to be taken for months versus an intravenous antibiotic to be administered for 7 days by the nurse in the intensive care unit. Therefore, future research should test whether our study findings can be applied to non-antibiotic trials.

## Conclusions

Our systematic review of antibiotic non-inferiority trials showed that ITT analysis on average produced wider CIs and was more conservative than PP analysis. Given that ITT is less prone to bias when an appropriate method for handling missing data is used, reporting of ITT analysis should be mandatory and ITT analysis should be the primary or co-primary analysis for non-inferiority trials on antibiotics.

## Supplementary Information

**Additional file 1.**

**Additional file 2.**

## Data Availability

All data generated or analysed during this study are included in this published article [and its supplementary information files].

## Abbreviations

ARR: Absolute risk reduction
CI: Confidence interval
EMA: European medicines agency
FDA: Food and drug administration
IQR: Interquartile range
ITT: Intention-to-treat
PP: Per protocol
RCT: Randomized controlled trial
