# Supplementary material for: Intention-to-treat analysis may be more conservative than per protocol analysis in antibiotic non-inferiority trials: a systematic review
Source: BMC Med Res Methodol. 2021 Apr 19;21:75. doi: 10.1186/s12874-021-01260-7 (PMC8054385; doi:10.1186/s12874-021-01260-7)
Supplement: Supplementary file 1 — Additional file 1. [file 12874_2021_1260_MOESM1_ESM.docx]

**Table of Contents**

Appendix Table 1. Description of each individual study ……………………………………………………………………………………………...…. Page 2 to 17

Appendix Table 2. Risk of bias assessment for each individual study …………………………………………………………………………………. Page 18 to 25

References for Appendix Table 1 and 2 ……………………………………………………...…………………………………………………………. Page 26 to 45

Appendix Table 3. Comparison of ITT to PP CI using Newcombe method ……………………………………………………...…………………….……. Page 46

Appendix Table 4. Agreement between ITT and PP analyses for conclusion on non-inferiority ……………………………………………………....……. Page 47

Appendix Table 5. Multivariable linear regression of difference between ITT lower CI and PP lower CI weighted by sample size in ITT population….… Page 48

Appendix Figure 1. Forest plot of point estimate and CI for ITT and PP analyses …………………………………………..……………………...……..… Page 49

Appendix Figure 2. Point estimate and lower CI limit difference between ITT and PP analyses …………………………………………..……………..… Page 50

Appendix Figure 3. ARR of excluded versus per protocol population ……………………………………………………..………………………………… Page 51

Appendix Figure 4. Success rate of treatment and control arms combined for excluded versus per protocol population …………………………………… Page 52

Appendix Figure 5. Success rate of treatment arm for excluded versus per protocol population …………………………………………………………..… Page 53

Appendix Figure 6. Success rate of control arm for excluded versus per protocol population …………………………………………………………….… Page 54

Appendix Figure 7. Funnel plot of both ITT and PP analyses ……………………………………………………..……………………….………………… Page 55

Appendix Figure 8. Funnel plot for only ITT analyses including excluded studies …………………………………………………………………..……… Page 56

Appendix Figure 9. Funnel plot for only PP analyses including excluded studies ……… …………………………………………………………..……… Page 57

Appendix Text 1. PRISMA checklist ……………………………………………………..…………………………...…………….………..………… Page 58 to 59

Appendix Text 2. Literature search strategy ……………………………………………………..…………………………...………………..……….. Page 60 to 65

**Appendix Table 1. Description of each individual study**

| Author  Publication year  Reference | Study period  Study location  Population | Study design | Infection syndrome | Treatment arms  N for PP / ITT | Primary outcome  Non-inferiority margin  Results^a^ | A) Author’s conclusion  B) Conclusion based on data  Other notes |
| --- | --- | --- | --- | --- | --- | --- |
| Nieuwkoop et al.  2017  [1] | 2008-2013  Multicenter  Adults | Double blinded | Febrile urinary tract infection | 7 days of antibiotics (N=92/94)  14 days of antibiotics (N=92/99) | Clinical cure  NIM = 10%  ITT: -4.5 90% CI -10.7 to 1.7  PP: -4.3 90% CI -1.02 to 0.07 | A) Inconclusive  B) Inconclusive |
| Chosidow et al.  2005  [2] | 2001-2002  Multicenter  Children & adults | Open label | Superficial pyodermas | Cloxacillin PO (N=162/171)  Pristamycin PO (N=149/163) | Cure rates  NIM = 15%  ITT: -2.1 95% CI -10.4 to 6.2  PP: -3.1 95% CI -11.3 to 5.1 | A) Non-inferiority proven  B) Non-inferiority proven |
| Harbarth et al.  2015  [3] | 2009-2013  Single center  Adults | Open label | MRSA infection | Trimethoprim-Sulfamethoxazole PO/IV plus Rifampin PO (N=59/75)  Linezolid PO/IV (N=66/75) | Clinical success  NIM = 20%  ITT: 4 95% CI -9.7 to 17.6  PP: 6.3 95% CI -6.8 to 19.2 | A) Non-inferiority proven  B) Non-inferiority proven |
| Brack et al.  2012  [4] | 2004-2007  Multicenter  Children | Open label | Febrile neutropenia | Amoxicillin PO and Ciprofloxacin PO (N=25/27)  Continue IV antibiotics (N=31/34) | Efficacy  NIM = 10%  ITT: 8.7 95% CI -9.5 to ∞  PP: 18 95% CI -0.4 to ∞ | A) Non-inferiority proven  B) Non-inferiority proven  Safety not assessable. |
| Goyal et al.  2018  [5] | 2012-2016  Multicenter  Children | Double blind | Bronchiectasis exacerbation | Azithromycin PO (N=73/82)  Amoxicillin-Clavunate PO (N=87/97) | Resolution of exacerbation  NIM = 20%  ITT: -0.5 95% CI -12.9 to 11.9  PP: -0.3 95% CI -11.8 to 11.1 | A) Non-inferiority proven  B) Non-inferiority proven |
| Bernard et al.  2002  [6] | 1998-2000  Multicenter  Adults | Open label | Erysipelas | Pristinamycin PO (N=102/138)  Penicillin PO (N=102/150) | Cure rates  NIM = 10%  ITT: 12 97% CI 1.7 to ∞  PP: 14.7 97% CI 3.3 to ∞ | A) Superiority proven  B) Superiority proven |
| Liu et al.  2019  [7] | 2009-2012  Multicenter  Adults | Open label | Hospital acquired and healthcare associated pneumonia | Cefoperazone-Sulbactam IV (N=66/71)  Cefepime IV (N=81/83) | Clinical success  NIM = 20%  ITT: 3 95% CI -9 to 15  PP: 2.8 95% CI -11.4 to 17 | A) Non-inferiority proven  B) Non-inferiority proven |
| Rudrabhatla et al.  2018  [8] | 2015-2016  Single center  Adults | Open label | Pyelonephritis | 7 days of antibiotics (N=26/27)  14 days of antibiotics (N=27/27) | Recurrence  NIM = 15%  ITT: 3.7 90% CI -6.15 to 15.01  PP: 3.85 90% CI -6.04 to 15.53 | A) Non-inferiority proven  B) Non-inferiority proven |
| Bernard et al.  2015  [9] | 2006-2011  Multicenter  Adults | Open label | Vertebral osteomyelitis | 6 weeks of antibiotics (N=146/176)  12 weeks of antibiotics (N=137/175) | Clinical cure  NIM = 10%  ITT: 0.05 95% CI -6.2 to 6.3  PP: -2.5 95% CI -8.2 to 2.9 | A) Non-inferiority proven  B) Non-inferiority proven |
| Cornely et al.  2012  [10] | 2007-2009  Multicenter  Adults age >=16 | Double blinded | C difficile colitis | Fidaxomicin PO (N=216/252)  Vancomycin PO (N=235/257) | Clinical cure  NIM = 10%  mITT: 0.9 95% CI -4.9 to 6.7  PP: 1.1 95% CI -4.3 to ∞ | A) Non-inferiority proven  B) Non-inferiority proven |
| Mitja et al.  2012  [11] | 2010-2011  Single center  Children | Open label | Yaws | Azithromycin PO (N=124)  Penicillin IM (N=126) | Serologic and clinical cure  NIM = 10%  ITT: 2.2 95% CI -6.8 to 11.1  PP: 3.4 95% CI -2.4 to 9.3 | A) Non-inferiority proven  B) Non-inferiority proven |
| Mikamo et al.  2018  [12] | 2014-2016  Multicenter  Adults | Double blinded | C difficile colitis | Fidaxomicin PO (N=85/104)  Vancomycin PO (N=95/108) | Global cure  NIM =10%  ITT: 1.2 95% CI -11.3 to 13.7  PP: 3.9 95% CI -9.1 to 16.8 | A) Inconclusive  B) Inconclusive |
| Snyman et al.  2009  [13] | 2007  Multicenter  Adults | Single blind | Respiratory tract infections | Generic Clarithromycin PO (N=141)  Non-generic Clarithromycin PO (N=136) | Clinical cure  NIM = 10%  ITT: not reported  PP: -1.6 CI not reported | A) Non-inferiority proven  B) Inconclusive |
| Malfertheiner et al.  2011  [14] | 2008-2009  Multicenter  Adults | Open label | H pylori infection | Quadruple therapy (N=178/216)  Triple therapy (N=161/222) | Eradication by urease test  NIM = 10%  ITT: 25 95% CI 15.5 to 33.3  PP: 23 95% CI 15.1 to 32.3 | A) Superiority proven  B) Superiority proven |
| Barrera et al.  2016  [15] | 2013-2014  Multicenter  Adults | Double blinded | Community acquired pneumonia | Solithromycin PO (N=403/426)  Moxifloxacin PO (N=407/434) | Early clinical response  NIM = 10%  ITT: 0.29 95% CI -5.5 to 6.1  PP: -0.19 95% CI -5.8 to 5.5 | A) Non-inferiority proven  B) Non-inferiority proven |
| Dalen et al.  2018  [16] | 2010-2014  Multicenter  Adults | Double blinded | Uncomplicated skin and soft tissue infection | Cephalexin PO (N=96/101)  Cefazolin IV and Probenecid PO (N=99/102) | Treatment failure  NIM = 10%  ITT: 0.1 90% -6.7 to 6.9  PP: -1.9 90% CI -3.7 to 7.6 | A) Non-inferiority proven  B) Non-inferiority proven |
| Bowen et al.  2014  [17] | 2009-2012  Multicenter  Children | Open label | Impetigo | Co-trimoxazole PO for 3 or 5 days (N=310/334)  Penicillin IM (N=146/156) | Treatment success  NIM = 10%  ITT: -0.5 95% CI -7.3 to 6.2  PP 0.2 95% CI -5.6 to 6.1 | A) Non-inferiority proven  B) Non-inferiority proven |
| Chen et al.  2016  [18] | 2014-2015  Single center  Adults | Open label | H pylori infection | Amoxicillin PO in quadruple therapy (N=142/156)  Tetracycline PO in quadruple therapy (N=128/156) | Eradication by urea breath test  NIM = 10%  ITT: 1.3 95% CI -4.8 to 7.4  PP: -1.7 95% CI -6.2 to 2.9 | A) Non-inferiority proven  B) Non-inferiority proven |
| Zhong et al.  2015  [19] | 2011-2013  Multicenter  Adults | Double blinded | Community acquired pneumonia | Ceftaroline IV (N=258/381)  Ceftriaxone IV (N=240/382) | Clinical cure  NIM = 10%  ITT: 13 95% CI 6.8 to 19.2  PP: 9.9 95% CI 2.8 to 17.1 | A) Superiority proven  B) Superiority proven |
| Stahlgren et al.  2019  [20] | 2015-2018  Multicenter  Children & adults | Open label | Streptococcus pharyngitis | Penicillin PO 4 times daily for 5 days (N=202/212)  Penicillin PO 3 times daily for 10 days (N=195/210) | Clinical cure  NIM = 10%  ITT: -4.2 95% CI -9.9 to 1.5  PP: -3.7 95% CI -9.7 to 2.2 | A) Non-inferiority proven  B) Non-inferiority proven |
| Chandra et al.  2008  [21] | 2004-2005  Multicenter  Children & adults | Open label | Intra-abdominal infection | Cefoperazone-sulbactam IV (N=136/154)  Ceftazidime IV, Amikacin IV and Metronidazole IV (N=132/152) | Clinical resolution  NIM = 12.5%  ITT: 10.1 95% CI 2 to 18.3  PP: 10.1 95% CI 2.1 to 18.1 | A) Superiority proven  B) Superiority proven |
| Pullman et al.  2017  [22] | 2013-2014  Multicenter  Adults | Double blinded | Acute bacterial skin and skin structure infections | Delafloxacin IV (N=294/331)  Vancomycin IV and Aztreonam IV (N=297/329) | Early clinical response  NIM = 10%  ITT: -2.6 95% CI -8.78 to 3.57  PP: -1.5 95% CI -7.2 to 4.18 | A) Non-inferiority proven  B) Non-inferiority proven |
| Wagenlehner et al.  2015  [23] | 2011-2013  Multicenter  Adults | Double blinded | Complicated urinary tract infection or pyelonephritis | Ceftolozane-Tazobactam IV (N=341/398)  Levofloxacin IV (N=353/402) | Composite cure based on clinical cure and bacterial eradication  NIM = 10%  ITT: 8.5 95% CI 2.3 to 14.6  PP: 8 95% CI 2 to 14 | A) Superiority proven  B) Superiority proven |
| Gerding et al.  2019  [24] | 2014-2017  Multicenter  Adults | Double blinded | C difficile colitis | Cadazolid PO (N=282/302 in IMPACT 1, N=288/318 in IMPACT 2)  Vancomycin PO (N=247/290 in IMPACT 1, N=259/301 in IMPACT 2) | Clinical cure  NIM = 10%  IMPACT 1  ITT: -1.4 95% CI -7.2 to 4.3  PP: -4.1 95% CI -9.2 to 1  IMPACT 2  ITT: -4.7 95% CI -10.7 to 1.3  PP: -4.9 95% CI -9.2 to 1 | IMPACT 1  A) Non-inferiority proven  B) Non-inferiority proven  IMPACT 2  A) Inconclusive  B) Inconclusive |
| Paul et al.  2015  [25] | 2007-2014  Multicenter  Adults | Open label | MRSA infection | High dose Trimethoprim-Sulfamethoxazole IV or PO (N=135)  Vancomycin IV (N=117) | Treatment failure at day 7  NIM = 15%  ITT: -11 95% CI -21.5 to 1.2  PP:7 CI not reported | A) Inconclusive  B) Inconclusive  Difference in failure highest in bacteremia for Trimethoprim-Sulfamethoxazole. |
| Nathan et al.  2005  [26] | 2003  Multicenter  Children & adults | Open label | Meningococcal meningitis | Ceftriaxone IM single dose (N=160/247)  Chloramphenicol IM (N=148/256) | Treatment failure at 72 hours  NIM = 10%  ITT: -0.3 90% CI -4.5 to 3.8  PP: 0.4 95% CI -3.8 to 4.6 | A) Non-inferiority proven  B) Non-inferiority proven |
| Rhee et al.  2015  [27] | 2012-2014  Multicenter  Adults | Double blinded | COPD exacerbation | Zabofloxacin PO for 5 days (N=143/175)  Moxifloxacin PO for 7 days (N=131/167) | Clinical cure  NIM = 10%  ITT: -0.1 95% CI -9 to 8.8  PP: 0.4 95% CI -7.7 to 8.6 | A) Non-inferiority proven  B) Non-inferiority proven |
| Llor et al.  2017  [28] | 2013-2016  Multicenter  Adults | Double blinded | Community acquired pneumonia | Penicillin PO (N=11/14)  Amoxicillin PO (N=25/25) | Clinical cure at 14 days  NIM = 15%  ITT: -28.6 95% CI -58.1 to -7.3  PP: -9.1 95% CI -41.3 to 6.4 | A) Inconclusive  B) Inconclusive  Stopped early. Trend favoring Amoxicillin PO. |
| Xu et al.  2016  [29] | 2011-2013  Multicenter  Adults | Double blinded | Moderate to severe diabetic foot infection | Ertapenem IV (N=219/267)  Piperacillin-Tazobactam IV (N=224/266) | Clinical response  NIM = 15%  ITT: -1.9 95% CI -7.3 to 3.3  PP: -3.8 95% CI -8.3 to 0 | A) Non-inferiority proven  B) Non-inferiority proven  Ertapenem had markedly lower rate of clinical resolution in severe diabetic foot infections |
| Dryden et al.  2016  [30] | 2012-2014  Multicenter  Adults | Double blinded | Complicated skin and soft tissue infection | Ceftaroline 600mg IV Q8H (N=395/506)  Vancomycin IV and Aztreonam IV (N=211/255) | Clinical cure  NIM = 10%  mITT: -0.95 95% CI -6.9 to 5.41  PP: 1.27 95% CI -4.32 to 7.48 | A) Non-inferiority proven  B) Non-inferiority proven |
| Ren et al.  2017  [31] | 2012-2014  Multicenter  Adults | Open label | Complicated urinary tract infection and acute pyelonephritis | Levofloxacin 750mg IV daily for 5 days (N=145/159)  Levofloxacin 500mg IV daily for 7-14 days (N=145/158) | Clinical success  NIM = 15%  ITT: 0.56 95% CI -6.16 to 7.29  PP: 2.76 95% CI -3.58 to 9.09 | A) Non-inferiority proven  B) Non-inferiority proven |
| Heystek et al.  2009  [32] | 1997-1998  Multicenter  Adults | Double blinded | Acute, uncomplicated pelvic inflammatory disease | Moxifloxacin PO (N=232/343)  Ciprofloxacin PO, Doxycycline PO, and Metronidazole PO (N=202/326) | Clinical success  NIM = 10%  ITT: 0.3 95% CI -5.8 to 6.9  PP: 1.4 95% CI -4.5 to 1.6 | A) Non-inferiority proven  B) Non-inferiority proven |
| Bocquet et al.  2012  [33] | 2004-2008  Multicenter  Children | Open label | Acute pyelonephritis | Cefixime PO for 10 days (N=52/61)  Ceftriaxone IV for 4 days then Cefixime PO for 6 days (N=44/58) | Renal scarring on follow-up scintigraphy  NIM = 10%  ITT: -3.8 95% CI -21.6 to 13.9  PP: 3.5 95% CI -14.7 to 21.7 | A) Inconclusive  B) Inconclusive |
| Bartacek et al.  2009  [34] | 2003-2004  Multicenter  Children & adults | Open label | Active pulmonary tuberculosis | Fixed dose Isoniazid, Rifampicin, Pyrazinamide and Ethambutol PO (N=412/582)  Single tablets of Isoniazid, Rifampicin, Pyrazinamide and Ethambutol PO (N=422/577) | Smear conversion  NIM = 10%  ITT: -2.26 95% CI -6.72 to 2.21  PP: -0.51 95% CI -2.27 to 1.23 | A) Non-inferiority proven  B) Non-inferiority proven |
| Torres et al.  2018  [35] | 2013-2015  Multicenter  Adults | Double blinded | Nosocomial pneumonia | Ceftazidime-Avibactam IV (N=257/401)  Meropenem IV (N=370/401) | Clinical cure  NIM = 12.5%  ITT: -4.2% 95% CI -10.8 to 2.5  PP: -0.7% 95% CI -7.86 to 6.39 | A) Non-inferiority proven  B) Non-inferiority proven |
| Gyssens et al.  2011  [36} | 2006-2008  Multicenter  Adults | Double blinded | Complicated skin and skin structure infections | Moxifloxacin IV then PO (N=361/426)  Piperacillin-Tazobactam IV then Amoxicillin-Clavunate PO (N=307/377) | Clinical success  NIM = 10%  ITT: 1.25 95% CI -3.8 to 6.3  PP: -0.72 95% CI -5.3 to 3.9 | A) Non-inferiority proven  B) Non-inferiority proven |
| Zhao et al.  2016  [37] | 2004-2005  Multicenter  Adults | Open label | Community acquired pneumonia | Levofloxacin 750mg IV daily for 5 days (N=208/221)  Levofloxacin 500mg IV daily for 7-14 days (N=219/227) | Clinical cure or improvement  NIM = 10%  ITT: -2.87 95% CI -7.64 to 1.9  PP: -2.14 95% CI -6.35 to 2.07 | A) Non-inferiority proven  B) Non-inferiority proven |
| Tanaseanu et al.  2009  [38] | 2004-2005  Multicenter  Adults | Double blinded | Community acquired pneumonia | Tigecycline IV (N=144/203)  Levofloxacin IV (N=136/200) | Clinical response  NIM = 15%  ITT: 2 95% CI -5.5 to 9.6  PP: 3.6 95% CI -4.5 to 11.8 | A) Non-inferiority proven  B) Non-inferiority proven |
| Towfigh et al.  2010  [39] | 2005-2008  Multicenter  Adults | Open label | Complicated intra-abdominal infection | Tigecycline IV (N=189/228)  Ceftriaxone IV and Metronidazole IV (N=187/220) | Clinical response  NIM = 15%  ITT: -7 95% CI -15.8 to 1.08  PP: -4 95% CI -13.1 to 5.1 | A) Non-inferiority proven  B) Inconclusive (ITT results crossed NIM) |
| Yakovlev et al.  2006  [40] | 1999-2002  Multicenter  Adults | Double blinded | Pneumonia acquired in skilled-care facilities or hospital outside ICU | Ertapenem IV (N=93/144)  Cefepime IV (N=102/146) | Clinical resolution or improvement  NIM = 15%  ITT: 4.1 95% CI -6.7 to 15  PP: 1.3 95% CI -9.4 to 11.8 | A) Non-inferiority proven  B) Non-inferiority proven |
| Nakane et al.  2015  [41] | 2006-2010  Multicenter  Adults | Open label | Febrile neutropenia | Cefozopran IV (N=90)  Meropenem IV (N=92)  Imipenem-Cilastatin IV (N=88)  Cefepime (N=85) | Clinical response  NIM = 10%  Number of successes reported, but risk difference and CI not reported | A) Inconclusive  B) Inconclusive |
| Waele et al.  2013  [42] | ?  Multicenter  Adults | Double blinded | Complicated intra-abdominal infection | Moxifloxacin IV (N=352/408)  Ertapenem IV (N=347/390) | Clinical success  NIM = 10%  ITT: -5 95% CI -9.9 to 0  PP: -3.9 95% CI -7.9 to 0.4 | A) Non-inferiority proven  B) Inconclusive (ITT showed inferiority) |
| Namias et al.  2007  [43] | 2001-2004  Multicenter  Adults | Double blinded | Complicated intra-abdominal infection | Ertapenem IV (N=123/189)  Piperacillin-Tazobactam IV (N=108/187) | Clinical cure  NIM = 15%  ITT: 2.3 95% CI -7.5 to 12  PP: 0.3 95% CI -9.6 to 10.5 | A) Non-inferiority proven  B) Non-inferiority proven |
| Lora-Tamayo et al.  2016  [44] | 2009-2013  Multicenter  Adults | Open label | Staphylococcal prosthetic joint infection | 8 weeks of Levofloxacin plus Rifampin PO (N=24/30)  Standard duration of Levofloxacin PO plus Rifampin PO (N=20/33) | Clinical cure  NIM = 15%  ITT: 15.7 95% CI -7.8 to 39.2  PP: -3.3 95% CI -18.3 to 11.7 | A) Non-inferiority proven  B) Inconclusive (PP results crossed NIM)  Stopped early due to slow recruitment. |
| Ibrahim et al.  2019  [45] | 2015-2017  Single center  Children | Open label | Moderate to severe cellulitis | Ceftriaxone IV at home (N=89/93)  Cloxacillin IV in hospital (N=91/95) | Treatment failure  ITT: 5.3 95% CI -0.8 to 11.3  PP: 6.5 95% CI 0.7 to 12.4 | A) Non-inferiority proven  B) Non-inferiority proven  No difference in acquisition of resistance organisms. |
| Solomkin et al.  2009  [46] | 2005-2007  Multicenter  Adults | Double blinded | Complicated intra-abdominal infection | Moxifloxacin IV (N=180)  Ceftriaxone IV and Metronidazole IV (N=181) | Clinical success  NIM = 15%  ITT: -4 95% CI -10.7 to 1.9  PP: -6.3 95% CI -11.7 to -1.7 | A) Non-inferiority proven  B) Inferiority proven in PP |
| Lv et al.  2017  [47] | 2014-2016  Multicenter  Adults | Double blinded | Acute bacterial skin and skin structure infection | Tedizolid phosphate IV then PO for 6 days (N=219/300)  Linezolid IV or PO for 10 days (N=231/298) | Early clinical response  NIM = 10%  ITT: -4.6 95% CI -11.2 to 2.2  PP: -2.1 95% CI -7.4 to 3.2 | A) Inconclusive  B) Inconclusive |
| Yuan et al.  2018  [48] | 2011-2012  Multicenter  Adults | Double blinded | Community acquired pneumonia | Nemonoxacin PO (N=305/328)  Levofloxacin PO (N=147/160) | Clinical cure  NIM = 10%  ITT: 0.9 95% CI -3.8 to 5.5  PP: 1.2 95% CI -3.4 to 5.8 | A) Non-inferiority proven  B) Non-inferiority proven |
| Liou et al.  2018  [49] | 2015-2017  Multicenter  Adults | Open label | H pylori infection | 14 days sequential therapy (N=297/310)  10 days quadruple therapy (N=290/310) | Eradication rate  NIM = 5%  ITT: -0.3 95% CI -4.7 to 4.4  PP: -1.6 95% CI -5.2 to 2 | A) Non-inferiority proven  B) Inconclusive (PP results cross NIM) |
| Aseffa et al.  2016  [50] | 2007-2011  Multicenter  Adults | Single blinded | Active pulmonary tuberculosis | Fixed dose combination of Isoniazid, Rifampicin, Ethambutol and Pyrazinamide (N=363/459)  Loose formulation combination of Isoniazid, Rifampicin, Ethambutol and Pyrazinamide (N=356/465) | Microbiologic cure  NIM = 4%  ITT: 1.55 90% CI -2.22 to 5.32  PP: 2 90% CI 0.13 to 3.8 | A) Non-inferiority proven  B) Non-inferiority proven |
| File et al.  2001  [51] | ?  Multicenter  Adults | Double blinded | Community acquired pneumonia | Gemifloxacin PO (N=216/290)  Trovafloxacin PO (N=207/281) | Clinical success  NIM = 10%  ITT: 6.4 95% CI 0.5 to 12.4  PP: 4.1 95% CI -1.1 to 9.3 | A) Non-inferiority proven  B) Non-inferiority proven |
| Harris et al.  2018  [52] | 2014-2017  Multicenter  Adults | Open label | ESBL E coli or Klebsiella pneumonia bloodstream infection | Piperacillin-Tazobactam IV (N=170/187)  Meropenem IV (N=186/191) | Mortality  NIM = 5%  ITT: -8.6 97.5% CI -14.5 to ∞  PP: -6.8 97.5% CI -12.8 to ∞ | A) Inconclusive  B) Inconclusive  Stopped early due to possible harm on interim analysis |
| Aliberti et al.  2017  [53] | 2012-2014  Multicenter  Adults | Open label | Community acquired pneumonia | Stop antibiotics 2 days after clinical stability (N=125)  Standard antibiotic duration based on physician decision (N=135) | Composite outcome of early failure  NIM = 5%  Number of successes reported, but risk difference and CI not reported | A) Inconclusive  B) Inconclusive  Stopped early due to possible harm on interim analysis |
| GIusti et al.  2016  [54] | ?  Multicenter  Adults | Single blinded | COPD exacerbation | Prulifloxacin PO (N=130)  Levofloxacin PO (N=128) | Therapeutic success  NIM = 15%  Number of successes reported, but risk difference and CI not reported | A) Non-inferiority proven  B) Inconclusive (CI not reported) |
| Oliva et al.  2005  [55] | 2002-2004  Multicenter  Adults | Double blinded | Complicated intra-abdominal infection | Tigecycline IV (N=247/309)  Imipenem-Cilastatin IV (N=255/312) | Clinical cure  NIM = 15%  ITT: -4.3 95% CI -11 to 2.5  PP: -1.7 95% CI -8.4 to 5.1 | A) Non-inferiority proven  B) Non-inferiority proven |
| Judlin et al.  2010  [56] | 2007-2008  Multicenter  Adults | Double blinded | Uncomplicated pelvic inflammatory disease | Moxifloxacin PO (N=194/228)  Levofloxacin PO and Metronidazole PO (N=190/232) | Clinical response at test-of-cure  NIM = 15%  ITT: -2.2 95% CI -10.1 to 5.9  PP: -3.2 95% CI -10.7 to 4.9 | A) Non-inferiority proven  B) Non-inferiority proven |
| Zhang et al.  2015  [57] | 2014  Single center  Adults | Open label | H pylori infection | Quadruple therapy with Amoxicillin PO and Metronidazole PO (N=97/108)  Quadruple therapy with Amoxicillin PO and Clarithromycin PO (N=98/107) | Eradication  NIM = 10%  ITT: 0.1 95% CI -7 to 7.2  PP: 2 95% CI -2.7 to 6.7 | A) Non-inferiority proven  B) Non-inferiority proven |
| Ross et al.  2006  [58] | 2003-2004  Multicenter  ? | Double blinded | Uncomplicated pelvic inflammatory disease | Moxifloxacin PO (N=275/378)  Oxofloxacin PO and Metronidazole PO (N=289/363) | Clinical resolution  NIM = 10%  ITT: -6.9 95% CI -12.3 to -0.9  PP: -0.5 95% CI -5.7 to 4 | A) Non-inferiority proven  B) Inferiority proven in ITT |
| Nicholson et al.  2012  [59] | 2006-2007  Multicenter  Adults | Double blinded | Community acquired pneumonia | Ceftobiprole IV (N=231/314)  Ceftriaxone IV (N=238/324) | Clinical cure  NIM = 10%  ITT: -2.9 95% CI -9.3 to 3.6  PP: -0.8 95% CI -6.9 to 5.3 | A) Non-inferiority proven  B) Non-inferiority proven |
| Wilson et al.  2012  [60] | ?  Multicenter  Adults | Double blinded | COPD exacerbation | Moxifloxacin PO (N=538/677)  Amoxicillin-Clavunate PO (N=518/675) | Clinical failure  ITT: 1.2 95% CI -3.03 to 5.5  PP: 1.4 95% CI -3.83 to 5.89 | A) Non-inferiority proven  B) Non-inferiority proven |
| Rob et al.  2019  [61] | 2016-2019  Single center  Adults | Open label | Rectal and pharyngeal gonorrhea | Gentamicin IM plus Azithromycin PO (N=72/73)  Ceftriaxone IM plus Azithromycin PO (N=71/72) | Microbiologic eradication  NIM = 7%  ITT: 0.02 95% CI -6.1 to 6.2  PP: 0 95% CI -5.1 to 5.1 | A) Non-inferiority proven  B) Non-inferiority proven |
| Petitpretz et al.  2007  [62] | 2003-2004  Multicenter  Adults | Open label | Acute exacerbation of chronic obstructive bronchitis | Levofloxacin PO (N=259/289)  Cefuroxime PO (N=258/296) | Clinical cure  NIM = 10%  mITT: 2.3 95% CI -1.8 to 6.2  PP: 0.8 95% CI -3.2 to 4.8 | A) Non-inferiority proven  B) Non-inferiority proven |
| Moussaoui et al.  2006  [63] | 2000-2003  Multicenter  Adults | Double blinded | Community acquired pneumonia | Amoxicillin IV for 3 days (N=54/56)  Amoxicillin IV then PO for total of 8 days (N=60/63) | Clinical success at day 10  NIM = 10%  ITT: 0.4 95% CI -11 to 12  PP: 0.1 95% CI -9 to 10 | A) Non-inferiority proven  B) Inconclusive on ITT analysis |
| Fabian et al.  2005  [64] | 2001-2003  Multicenter  Children & adults | Double blinded | Complicated skin and skin structure infection | Imipenem-cilastatin IV (N=261/510)  Meropenem IV (N=287/527) | Clinical response  NIM = 10%  ITT: -3.8 95% CI -9.8 to 2.1  PP: 3.3 95% CI -2.8 to 9.3 | A) Non-inferiority proven  B) Non-inferiority proven |
| Siquier et al.  2006  [65] | 2001-2003  Multicenter  Adults | Double blinded | Community acquired pneumonia | Amoxicillin-Clavunate PO BID (N=288/374)  Amoxicillin-Clavunate PO TID (N=148/192) | Clinical success  NIM = 10%  ITT: 1.4 95% CI -5.2 to 8  PP: 1.1 95% CI -4.4 to 6.6 | A) Non-inferiority proven  B) Non-inferiority proven |
| Corey et al.  2010  [66] | 2007  Multicenter  Adults | Double blinded | Complicated skin and skin structure infection | Ceftaroline IV (N=316/351)  Vancomycin IV and Aztreonam IV (N=300/347) | Clinical cure  NIM = 10%  ITT: 1 95% CI -4.2 to 6.2  PP: -2.2 95% CI -6.6 to 2.1 | A) Non-inferiority proven  B) Non-inferiority proven |
| Wilcox et al.  2010  [67] | 2007  Multicenter  Adults | Double blinded | Complicated skin and skin structure infection | Ceftaroline IV (N=294/342)  Vancomycin IV and Aztreonam IV (N=292/338) | Clinical cure  NIM = 10%  ITT: -0.4 95% CI -5.8 to 5  PP: 0.1 95% CI -4.4 to 4.5 | A) Non-inferiority proven  B) Non-inferiority proven |
| File et al.  2007  [68] | 2004-2005  Multicenter  Adults | Double blinded | Community acquired pneumonia | Gemifloxacin PO for 5 days (N=242/256)  Gemifloxacin PO for 7 days (N=227/254) | Clinical success  NIM = 10%  ITT: 5.6 95% CI 0.34 to 10.81  PP: -2.9 95% CI -1.48 to 7.42 | A) Non-inferiority proven  B) Non-inferiority proven |
| File et al.  2011  [69] | 2008  Multicenter  Adults | Double blinded | Community acquired pneumonia | Ceftaroline IV (N=224/291)  Ceftriaxone IV (N=234/300) | Clinical cure  NIM = 10%  ITT: 6.2 95% CI -0.2 to 12.6  PP: 8.4 95% CI 1.4 to 15.4 | A) Non-inferiority proven  B) Non-inferiority proven |
| Low et al.  2011  [70] | 2007-2008  Multicenter  Adults | Double blinded | Community acquired pneumonia | Ceftaroline IV (N=235/289)  Ceftriaxone IV (N=215/273) | Clinical cure  NIM = 10%  ITT: 5.9 95% CI -1 to 12.7  PP: 4.9 95% CI -2.5 to 12.5 | A) Non-inferiority proven  B) Non-inferiority proven |
| Huang et al.  2018  [71] | 2016-2017  Multicenter  Adults | Double blinded | Acute bacterial skin and skin structure infection | Iclaprim IV (N=268/298)  Vancomycin IV (N=282/300) | Early clinical response  NIM = 10%  ITT: -0.13 95% CI -6.42 to 6.17  PP: -1.49 95% CI -7.44 to 4.46 | A) Non-inferiority proven  B) Non-inferiority proven |
| Corey et al.  2014  [72] | 2011-2012  Multicenter  Adults | Double blinded | Acute bacterial skin infection | Oritavancin IV once (N=394/475)  Vancomycin IV (N=397/479) | Early clinical response  NIM = 10%  ITT: 3.4 95% CI -1.6 to 8.4  PP: 1.2 95% CI -3.6 to 5.9 | A) Non-inferiority proven  B) Non-inferiority proven |
| Stets et al.  2019  [73] | 2015-2017  Multicenter  Adults | Double blinded | Community acquired pneumonia | Omadacycline IV then PO (N=356/386)  Moxifloxacin IV then PO (N=360/388) | Early clinical response  NIM = 10%  ITT: -1.6 95% CI -7.1 to 3.8  PP: -0.7 95% CI -5.7 to 4.3 | A) Non-inferiority proven  B) Non-inferiority proven |
| O’Riordan et al.  2019  [74] | 2016-2017  Multicenter  Adults | Double blinded | Acute bacterial skin and skin structure infections | Omadacycline PO (N=337/360)  Linezolid PO (N=360/360) | Early clinical response  NIM = 10%  ITT: 5 95% CI -0.2 to 10.3  PP: 2.1 95% CI -2 to 6.3 | A) Non-inferiority proven  B) Non-inferiority proven |
| Federico et al.  2012  [75] | 2011  Multicenter  Adults | Open label | H pylori infection | Concomitant therapy for 5 days (Esomeprazole, Amoxicillin, Levofloxacin, Tinidazole) (N=86/90)  Sequential therapy for 10 days (N=88/90) | Eradication  NIM = 10%  ITT: 1.1 95% CI -7.6 to 9.8  PP: -1 95% CI -8 to 5.9 | A) Non-inferiority proven  B) Non-inferiority proven |
| Freire et al.  2010  [76] | 2004-2006  Multicenter  Adults | Double blinded | Hospital acquired pneumonia | Tigecycline IV (N=268/440)  Imipenem-Cilastatin IV (N=243/429) | Clinical response  NIM = 15%  ITT: -4.8 95% CI -11 to 1.3  PP: -10.4 95% CI -17.8 to -3 | A) Inconclusive  B) Inferiority proven in PP analysis |
| Lauf et al.  2014  [77] | 2006-2009  Multicenter  Adults | Double blinded | Diabetic foot infection without osteomyelitis | Tigecycline IV (N=408/477)  Ertapenem IV +/- Vancomycin IV (N=405/466) | Clinical response  NIM = 10%  ITT: -6.7 95% CI -12.3 to -1.1  PP: -5.5 95% CI -11 to 0.1 | A) Inconclusive  B) Inferiority proven |
| Dartois et al.  2008  [78] | 2004-2005  Multicenter  Adults | Double blinded | Community acquired pneumonia | Tigecycline IV (N=125/173)  Levofloxacin IV (N=120/173) | Clinical response  NIM = 15%  ITT: 2.3 95% CI -6.1 to 10.8  PP: 3.8 95% CI -5.3 to 12.8 | A) Non-inferiority proven  B) Non-inferiority proven |
| Bergallo et al.  2009  [79] | 2003-2005  Multicenter  Adults | Double blinded | Community acquired pneumonia | Tigecycline IV (N=138/191)  Levofloxacin IV (N=156/203) | Clinical response  NIM = 15%  mITT: 0.2 95% CI -8.5 to 8.9  PP: 3.4 95% CI -4.4 to 11.2 | A) Non-inferiority proven  B) Non-inferiority proven |
| Sacchidanad et al.  2005  [80] | ?  Multicenter  Adults | Double blinded | Complicated skin and skin structure infections | Tigecycline IV (N=199/277)  Vancomycin IV and Aztreonam IV (N=198/160) | Clinical response  NIM = 15%  ITT: -1.5 95% CI -9 to 6.1  PP: 0.6 95% CI -7.4 to 8.6 | A) Non-inferiority proven  B) Non-inferiority proven |
| Louie et al.  2011  [81] | 2006-2008  Multicenter  Adults | Double blinded | C difficile colitis | Fidaxomicin PO (N=265/287)  Vancomycin PO (N=283309) | Clinical cure  NIM = 10%  mITT: 2.4 97.5% CI -3.1 to ∞  PP: 2.3 97.5% CI -2.6 to ∞ | A) Non-inferiority proven  B) Non-inferiority proven |
| Solomkin et al.  2015  [82] | 2011-2013  Multicenter  Adults | Double blinded | Complicated intra-abdominal infection | Ceftolozane-Tazobactam IV and Metronidazole IV (N=275/389)  Meropenem IV (N=321/417) | Clinical cure  NIM = 10%  ITT: -4.2 95% CI -8.91 to 0.54  PP: -1 95% CI -4.52 to 2.59 | A) Non-inferiority proven  B) Non-inferiority proven |
| Weiss et al.  2009  [83] | 2001-2002  Multicenter  Adults | Open label | Complicated intra-abdominal infection | Moxifloxacin IV then PO (N=246/289)  Ceftriaxone IV and Metronidazole IV, then Amoxicillin-Clavunate PO (N=265/295) | Clinical success  NIM = 10%  ITT: -5.3 95% CI -12.4 to 1.5  PP: -1.4 95% CI -8.9 to 4.2 | A) Non-inferiority proven  B) Inconclusive in ITT analysis |
| Molina-Infante et al.  2013  [84] | 2011-2012  Multicenter  Adults | Open label | H pylori infection | Hybrid quadruple therapy for total of 21 days (N=171)  Concomitant quadruple therapy for total of 14 days (N=172) | Eradication  NIM = 8%  Number of successes reported, but risk difference and CI not reported | A) Non-inferiority proven  B) Inconclusive |
| Mazuski et al.  2016  [85] | 2012-2014  Multicenter  Adults | Double blinded | Complicated intra-abdominal infection | Ceftazidime-Avibactam IV and Metronidazole IV (N=410/520)  Meropenem IV (N=416/523) | Clinical cure  NIM = 12.5  ITT: -2.4 95% CI -6.9 to 2.1  PP: -0.8 95% CI -4.61 to 2.89 | A) Non-inferiority proven  B) Non-inferiority proven |
| Chung et al.  2011  [86] | 2008-2010  Multicenter  Adults | Unclear | H pylori infection | Quadruple therapy for 7 days (N=98)  Quadruple therapy for 14 days (N=101) | Eradication  NIM = 15%  Number of successes reported, but risk difference and CI not reported | A) Non-inferiority proven  B) Inconclusive as no CI provided |
| Dore et al.  2011  [87] | 2008-2009  Single center  Adults | Open label | H pylori infection | Quadruple therapy for 10 days (N=215)  Quadruple therapy for 14 days (N=202) | Eradication  NIM = 10%  Number of successes reported, but risk difference and CI not reported | A) Non-inferiority proven  B) Inconclusive |
| Tsay et al.  2015  [88] | 2009-2010  Single center  Adults | Open label | H pylori infection | Sequential quadruple therapy (N=60)  Reverse sequential quadruple therapy (N=62) | Eradication  NIM = 10%  Number of successes reported, but risk difference and CI not reported | A) Non-inferiority proven  B) Inconclusive |
| Bohbot et al.  2010  [89] | 2007-2008  Multicenter  Adults | Double blinded | Bacterial vaginosis | Secnidazole PO once (N=216/290)  Metronidazole PO for 7 days (N=202/287) | Therapeutic success  NIM = 10%  ITT: 0.5 95% CI -7.6 to 8.5  PP: 0.5 95% CI -8.7 To 9.8 | A) Non-inferiority proven  B) Non-inferiority proven |
| Jindani et al.  2014  [90] | 2008-2011  Multicenter  Adults | Double blinded | Active pulmonary tuberculosis | 4 months therapy including Moxifloxacin PO (N=165/193)  6 month therapy including Moxifloxacin PO (N=186/212)  Standard 6 month therapy (N=163/188) | Unfavorable outcomes  NIM = 4%  4 months therapy including Moxifloxacin vs. standard 6 months therapy  ITT: -13.1 95% CI -19.4 to 6.8  PP: -13.6 95% CI -19.1 to 8.1  6 months therapy including Moxifloxacin vs. standard 6 months therapy  mITT: -0.4 95% CI -5.6 to 4.7  PP: 1.8 95% CI -2.4 to 6.1 | 4 months therapy including Moxifloxacin vs. standard 6 months therapy  A) Inconclusive  B) Inconclusive  6 months therapy including Moxifloxacin vs. standard 6 months therapy  A) Non-inferiority proven  B) Non-inferiority proven  Early stopping due to delayed start, slow recruitment |
| Lienhardt et al.  2011  [91] | 2003-2008  Multicenter  Adults | Open label | Active pulmonary tuberculosis | Fixed dose Rifampin PO, Isonidazid PO, Pyrazinamide PO and Ethambutol PO (N=591/684)  Separate Rifampin PO, Isonidazid PO, Pyrazinamide PO and Ethambutol PO (N=579/664) | Favorable outcome  NIM = 4%  ITT: -1.5 90% CI -4.7 to 1.8  PP: -0.7 90% -3 to 1.5 | A) Inconclusive  B) Inconclusive |
| Merle et al.  2014  [92] | 2005-2009  Multicenter  Adults | Open label | Active pulmonary tuberculosis | 4 months therapy including Gatifloxacin PO (N=651/694)  Standard 6 months therapy of Rifampin PO, Isonidazid PO, Pyrazinamide PO and Ethambutol PO (N=601/662) | Unfavorable outcome  NIM = 6%  ITT: -3.5 95% CI -7.7 to 0.7  PP: -5.5 95% CI -9.4 to -1.6 | A) Inconclusive  B) Inferiority proven in PP analysis |
| Gillespie et al.  2014  [93] | ?  Multicenter  Adults | Double blinded | Active pulmonary tuberculosis | 4 months therapy where Moxifloxacin PO replaces Ethambutol PO (N=514/568)  4 months therapy where Moxifloxacin PO replaces Isoniazid PO (N=524/551)  Standard 6 months therapy of Rifampin PO, Isonidazid PO, Pyrazinamide PO and Ethambutol PO (N=510/555) | Unfavorable outcome  NIM = 6%  4 months therapy where Moxifloxacin PO replaces Ethambutol PO vs. standard 6 months therapy  mITT: -7.8 97.5% CI -13 to 2.7  PP: -6.1 97.5% CI -10.5 to 1.7  4 months therapy where Moxifloxacin PO replaces Isoniazid PO vs. standard 6 months therapy  mITT: -9 97.5% CI -14.2 to -3.8  PP: -11.4 97.5% CI -16.1 to -6.7 | A) Inconclusive  B) Inconclusive |
| Nunn et al.  2019  [94] | 2012-2015  Multicenter  Adults | Open label | Rifampin resistant active tuberculosis | Short regimen (9-11 months) (N=227/245)  Long regimen (20 months) (N=83/124) | Favorable outcome  NIM = 10%  ITT: -1 95% CI -9.5 to 7.5  PP: 0.7 95% CI -9.1 to 10.5 | A) Non-inferiority proven  B) Non-inferiority proven |
| Kaye et al.  2018  [95] | 2014-2016  Multicenter  Adults | Double blinded | Complicated urinary tract infection | Meropenem-Varbobactam IV (N=272)  Piperacillin-Tazobactam IV (N=273) | Composite cure  NIM = 15%  mITT: 4.5 95% CI 0.7 to 9.1  PP: Number of successes reported, but risk difference and CI not reported | A) Non-inferiority proven  B) Non-inferiority proven |
| File et al.  2004  [96] | 2001-2002  Multicenter  Adults age >=16 | Double blinded | Community acquired pneumonia | Amoxicillin-Clavunate 2000/125mg PO BID (N=247/322)  Amoxicillin-Clavunate 875/125mg PO BID (N=226/311) | Clinical response  NIM = 10%  ITT: 7 95% CI 0.9 to 13  PP: 2.7 95% CI -3 to 8.3 | A) Non-inferiority proven  B) Non-inferiority proven |
| Van Rensburg et al.  2010  [97] | ?  Multicenter  Adults | Double blinded | Community acquired pneumonia | Nemonoxacin 750mg PO daily (N=82/89)  Nemonoxacin 500mg PO daily (N=79/86)  Levofloxacin 500mg PO daily (N=79/90) | Clinical response  NIM = 15%  Nemonoxacin 750mg PO daily vs. Levofloxacin  ITT: 2.6 97.5% CI -10.5 to 15.6  PP: 1.2 97.5% CI -12.1 to 14.6  Nemonoxacin 500mg PO daily vs. Levofloxacin  ITT: -4.7 97.5% CI -18.6 to 9.1  PP: -4.3 97.% CI -18.7 to 9.1 | Nemonoxacin 750mg PO daily vs. Levofloxacin  A) Non-inferiority proven  B) Non-inferiority proven  Nemonoxacin 500mg PO daily vs. Levofloxacin  A) Non-inferiority proven  B) Non-inferiority proven |
| Desrosiers et al.  2008  [98] | 2004-2005  Multicenter  Adults | Open label | Acute bacterial sinusitis | Telithromycin PO (N=123/144)  Amoxicillin-Clavunate PO (N=125/146) | Clinical success  NIM = 15%  ITT: -0.9 95% CI -9.2 to 7.5  PP: -0.2 95% CI -8.9 to 8.5 | A) Non-inferiority proven  B) Non-inferiority proven |
| Lucasti et al.  2008  [99] | 2004-2006  Multicenter  Adults | Double blinded | Complicated intra-abdominal infection | Doripenem IV (N=163/195)  Meropenem IV (N=156/190) | Clinical response  NIM = 15%  mITT: -1 95% CI -9.7 to 7.7  PP: 0.6 95% CI -7.7 to 9 | A) Non-inferiority proven  B) Non-inferiority proven |
| Corey et al.  2015  [100] | 2011-2013  Multicenter  Adults | Double blinded | Gram-positive acute bacterial skin and skin structure infections | Oritavancin IV once (N=427/503)  Vancomycin IV for 7-10 days (N=408/502) | Early clinical response  NIM = 10%  mITT: -2.7 95% CI -7.5 to 2  PP: -4.1 95% CI -8.9 to 6 | A) Non-inferiority proven  B) Non-inferiority proven |
| Clegg et al.  2006  [101] | 2001-2003  Single center  Children | Single blinded | Streptococcal pharyngitis | Amoxicillin PO daily (N=294/326)  Amoxicillin PO BID (N=296/326) | Bacteriologic failure  NIM = 10%  ITT: 0.3 90% CI -5.8 to 6.4  PP: -4.53 90% CI -9.7 to 0.6 | A) Non-inferiority proven  B) Non-inferiority proven |
| Zanetti et al.  2003  [102] | 1997-1999  Multicenter  Adults | Single blinded | Nosocomial pneumonia in ICU patients | Cefepime IV (N=108/132)  Imipenem-Cilastatin IV (N=101/138) | Clinical response  NIM = 15%  ITT: 2 95% CI -9 to 14  PP: -4 95% CI -16 to 8 | A) Inconclusive  B) Inconclusive |
| Rimoin et al.  2011  [103] | 2001-2003  Multicenter  Children | Open label | Streptococcal pharyngitis | Amoxicillin PO daily (N=54/64 in Croatia, 37/123 in Egypt)  Penicillin IM once (N=57/57 in Croatia, 124/124 in Egypt) | Bacteriologic eradication  NIM = 10%  Croatia group  ITT: 2.5 95% CI -13.8 to 18.9  PP: 1.1 95% CI -16.2 to 18.5  Egypt group  ITT: -15.1 95% CI -26.6 to -3.4  PP: -9.3 95% CI -26.3 to 7.8 | Croatia group:  A) Inconclusive  B) Inconclusive  Egypt group:  A) Inconclusive  B) Inconclusive  Stopped early due to loss to follow-up |
| Uranga et al.  2016  [104] | 2012-2013  Multicenter  Adults | Open label | Community acquired pneumonia | 5 days of antibiotics (N=162)  Antibiotic duration as determined by physicians (N=150) | Clinical success  NIM = 3%  ITT: 7.7 No CI provided  PP: 9.3 No CI provided | A) Non-inferiority proven  B) Inconclusive |
| Li et al.  2019  [105] | 2010-2015  Multicenter  Adults | Open label | Bone and joint infection | Oral antibiotics (N=466/527)  IV antibiotics (N=443/527) | Treatment failure at 1 year  NIM = 7.5%  ITT: -1.4 90% CI -4.9 to 2.2  PP: -2.5 90% CI -6.3 to 1.3 | A) Inconclusive  B) Inconclusive |
| Naber et al.  2004  [106] | 2001  Multicenter  Adults | Open label | Complicated urinary tract infection due to gram-positive bacteria | Daptomycin IV (N=29/29)  Ciprofloxacin IV (N=26/28) | Microbiologic efficacy  NIM = 10%  mITT: 4.2 95% CI -16.3 to 24.7  PP: -1.8 95% CI -21.4 to 17.7 | A) Inconclusive  B) Inconclusive  Stopped early due to slow recruitment |
| Noel et al.  2008  [107] | 2004-2005  Multicenter  Adults | Double blinded | Complicated skin and skin structure infection caused by gram positive bacteria | Ceftobiprole IV (N=282/397)  Vancomycin IV (N=277/387) | Clinical cure  NIM = 10%  ITT: 0.3 95% CI -5.5 to 6.1  PP: -0.2 95% CI -4.4 to 3.9 | A) Non-inferiority proven  B) Non-inferiority proven |
| UpChurch et al.  2006  [108] | 2000-2001  Multicenter  Adults | Double blinded | Acute bacterial sinusitis | Faropenem PO for 7 days (N=295/366)  Faropenem PO for 10 days (N=280/363)  Cefuroxime PO for 10 days (N=286/370) | Clinical response  NIM = 10%  Faropenem PO for 7 days vs. Cefuroxime:  ITT: 4 95% CI -2.7 to 10.5  PP: 5.8 95% CI 0.1 to 13.6  Faropenem PO for 10 days vs. Cefuroxime:  ITT: 2.6 95% CI -3.9 to 9.5  PP: 7.3 95% CI 1.7 to 15.2 | Faropenem PO for 7 days vs. Cefuroxime:  A) Non-inferiority proven  B) Non-inferiority proven  Faropenem PO for 10 days vs. Cefuroxime:  A) Non-inferiority proven  B) Non-inferiority proven |
| O’Riordan et al.  2018  [109] | 2014-2016  Multicenter  Adults | Double blinded | Acute bacterial skin and skin structure infection | Delafloxacin IV then PO (N=395/423)  Vancomycin IV and Aztreonam IV (N=387/427) | Early clinical response  NIM = 10%  ITT: 3.1 95% CI -2 to 8.3  PP; 3.1 95% CI -1.8 to 8 | A) Non-inferiority proven  B) Non-inferiority proven |
| Chastre et al.  2008  [110] | 2004-2006  Multicenter  Adults | Open label | Ventilator associated pneumonia | Doripenem IV (N=126/249)  Imipenem-Cilastatin IV (N=122/252) | Clinical cure  NIM = 20%  mITT: 1.2 95% CI -7.9 to 10.3  PP: 3.5 95% CI -9.1 to 16.1 | A) Non-inferiority proven  B) Non-inferiority proven |
| Leroy et al.  2005  [111] | 2000-2002  Multicenter  Adults | Open label | ICU patients with community acquired pneumonia | Levofloxacin IV (N=139/149)  Cefotaxime IV and Ofloxacin IV (N=132/159) | Clinical response  NIM = 15%  mITT: -2.2 95% CI -12.4 to 8.0  PP: -0.4 95% CI -10.8 to 10.0 | A) Non-inferiority proven  B) Non-inferiority proven |
| Torres et al.  2008  [112] | 2004-2005  Multicenter  Adults | Double blinded | Community acquired pneumonia requiring hospitalization | Moxifloxacin IV then PO (N=291/368)  Ceftriaxone IV and Levofloxacin IV then PO (N=278/365) | Clinical cure  NIM = 10%  ITT: -4.2 95% CI -9.7 to 1.4  PP: -3 95% CI -8.1 to 2.2 | A) Non-inferiority proven  B) Non-inferiority proven |
| Agweyu et al.  2015  [113] | 2011-2013  Multicenter  Children | Open label | Severe pneumonia | Amoxicillin PO (N=260/263)  Penicillin IM (N=261/263) | Treatment failure  NIM = 7%  ITT: 0.4 95% CI -4.2 to 5  PP: 0.3 95% CI -4.3 to 5 | A) Non-inferiority proven  B) Non-inferiority proven  Stopped early due to limited budget |
| Lojanapiwat et al.  2019  [114] | 2013-2015  Multicenter  Adults | Open label | Complicated urinary tract infection and acute pyelonephritis | Sitafloxacin PO (N=108/141)  Ceftriaxone IV then Cefdinir PO (N=103/148) | Clinical success  NIM = 15%  ITT: 2.8 95% CI -4.2 to 9.7  PP: -1.8 95% CI -5.9 to 1.9 | A) Non-inferiority proven  B) Non-inferiority proven |
| Iversen et al.  2019  [115] | 2011-2017  Multicenter  Adults | Open label | Left sided infective endocarditis | 10 days of IV, then PO antibiotics (N=197/201)  6 weeks of IV antibiotics (N=199/199) | Composite outcome of mortality, unplanned cardiac surgery, embolic event and relapse of bacteremia.  NIM = 10%  ITT: 3.1 95% CI -3.4 to 9.6  PP: 3 95% CI -3.2 to 9.2 | A) Non-inferiority proven  B) Non-inferiority proven |
| Solomkin et al.  2017  [116] | 2013-2014  Multicenter  Adults | Double blinded | Complicated intra-abdominal infections | Eravacycline IV (N=239/270)  Ertapenem IV (N=238/268) | Clinical cure  mITT: -1.8 95% CI -7.4 to 3.8  PP: -1.7 95% CI -6.3 to 2.8 | A) Non-inferiority proven  B) Non-inferiority proven |
| Awad et al.  2014  [117] | 2005-2007  Multicenter  Adults | Double blinded | Hospital acquired pneumonia | Ceftobiprole IV (N=251/391)  Linezolid IV and Ceftazidime IV (N=244/390) | Clinical cure  NIM = 15%  ITT: -2.9 95% CI -10 to 4.1  PP: -2 95% CI -10 to 6.1 | A) Non-inferiority proven  B) Non-inferiority proven  Signal for ham in ventilator associated pneumonia group. |
| Bradley et al.  2007  [118] | 2002-2004  Multicenter  Children | Open label | Community acquired pneumonia | Levofloxacin PO (N=405/504)  Standard therapy for community acquired pneumonia (N=134/170) | Clinical cure  NIM = 10%  mITT: 2 95% CI -4.1 to 8.1  PP: 0.3 95% CI -4.3 to 4.9 | A) Non-inferiority proven  B) Non-inferiority proven |
| Klausner et al.  2007  [119] | 2005-2006  Multicenter  Adults | Double blinded | Acute pyelonephritis | Levofloxacin IV or PO for 5 days (N=80/94)  Ciprofloxacin IV or PO for 10 days (N=76/98) | Microbiologic eradication  NIM = 15%  ITT: 3.4 95% CI -7.6 to 14.4  PP: -0.9 95% CI -8.9 to 7.1 | A) Non-inferiority proven  B) Non-inferiority proven |
| Fowler et al.  2006  [120] | 2002-2005  Multicenter  Adults | Open label | Staphylococcus aureus bacteremia | Daptomycin IV +/- Gentamicin IV (N=79/120)  Standard therapy plus Gentamicin IV (N=60/115) | Clnical success  NIM = 20%  ITT: 3.4 95% CI -8.9 to 15.7  PP: 1.1 95% CI -15.6 to 17.8 | A) Non-inferiority proven  B) Non-inferiority proven |
| Saar et al.  2019  [121] | 2016-2018  Single center  Adults | Open label | Appendicitis | 24 hours of antibiotics post-op (N=39)  >24 hours of antibiotics post-op (N=41) | Post-operative complications  NIM = ?  ITT: -11.4 CI not reported  PP: -7.4 CI not reported | A) Non-inferiority proven  B) Inconclusive, as NIM not provided |
| Fourcroy et al.  2005  [122] | 2003-2004  Multicenter  Adults | Double blinded | Uncomplicated urinary tract infection | Ciprofloxacin ER daily for 3 days (N=272/293)  Ciprofloxacin PO BID for 3 days (N=251/268) | Microbiologic eradication  NIM = 10%  mITT: 2.7 95% CI -3.16 to 8.56  PP: 3.8 95% CI -0.99 to 8.59 | A) Non-inferiority proven  B) Non-inferiority proven |
| Hooton et al.  2012  [123] | 2005-2009  Single center  Adults | Double blinded | Acute uncomplicated cystitis | Cefopoxodime PO (N=133/150)  Ciprofloxacin PO (N=135/150) | Clinical cure  NIM = 10%  ITT: -12 95% CI -21 to -3  PP: -12 95% CI -20 to -4 | A) Inconclusive  B) Inferiority proven |
| Noel et al.  2008  [124] | 2005-2006  Multicenter  Adults | Double blinded | Complicated skin and skin structure infections | Ceftobiprole IV (N=485/547)  Vancomycin IV and Ceftazidime IV (N=244/281) | Clinical cure  NIM = 10%  ITT: 1.1 95% CI -4.5 to 6.7  PP: 0.3 95% CI -4.2 to 4.9 | A) Non-inferiority proven  B) Non-inferiority proven |
| Peterson et al.  2008  [125] | ?  Multicenter  Adults | Double blinded | Complicated urinary tract infection or acute pyelonephritis | Levofloxacin 750mg PO or IV daily for 5 days (N=265/317)  Ciprofloxacin 400mg IV or 500mg PO BID for 10 days (N=241/302) | Microbiologic eradication  NIM = 15%  mITT: 0 95% CI -6.3 to 6.3  PP: 3.2 95% CI -2.5 to 8.9 | A) Non-inferiority proven  B) Non-inferiority proven |
| English et al.  2012  [126] | 2006-2007  Multicenter  Adults | Double blinded | Community acquired pneumonia | Cethromycin PO (442/518)  Clarithromycin PO (429/507) | Clinical cure  NIM = 15%  ITT: -1.8 95% CI -6.4 to 2.8  PP: -2.1 95% CI -5.4 to 1.2 | A) Non-inferiority proven  B) Non-inferiority proven |
| Rubinstein et al.  2011  [127] | 2005-2007  Multicenter  Adults | Double blinded | Hospital acquired pneumonia due to gram positive pathogens | Televancin IV (N=312/749)  Vancomycin IV (N=342/754) | Clinical cure  NIM = 20%  ITT: -0.7 95% CI -5.6 to 4.3  PP: 1.7 95% CI -4.3 to 7.7 | A) Non-inferiority proven  B) Non-inferiority proven |
| Mir et al.  2019  [128] | 2013-2017  Multicenter  Adults | Double blinded | Complicated urinary tract infection and acute pyelonephritis | Ceftriaxone-Sulbactam-Disodium EDTA IV (N=72/74)  Meropenem IV (N=68/69) | Clinical cure  NIM = 10%  ITT: 6 95% CI -2.6 to 16  PP: 7.4 95% CI 0.2 to 16.8 | A) Non-inferiority proven  B) Non-inferiority proven |
| Solomkin et al.  2019  [129] | 2016-2017  Multicenter  Adults | Double blinded | Complicated intra-abdominal infection | Eravacycline IV (N=174/195)  Meropenem IV (N=194/205) | Clinical cure  NIM = 12.5%  mITT: -0.5 95% CI -6.3 to 5.2  PP: -0.4 95% CI -4.9 to 3.8 | A) Non-inferiority proven  B) Non-inferiority proven |
| Chen et al.  2018  [130] | 2012-2015  Multicenter  Adults | Double blinded | Complicated intra-abdominal infection | Tigecycline IV (N=207/232)  Imipenem-Cilastatin IV (N=205/231) | Clinical response  NIM = 15%  ITT: -6 95% CI -12.8 to 0.8  PP: -6.7 95% CI -12 to -1.4 | A) Non-inferiority proven  B) Inferiority proven |
| Sapmaz et al.  2017  [131] | 2014  Single center  Adults | Open label | H pylori infection | Amoxicillin PO and high dose Rabeprazole PO (N=98)  Rabeprazole PO, Bismuth PO, Tetracycline PO, Metraonidazole PO (N=98) | Eradication  NIM = ?  ITT: 3.1 CI not reported  ITT: 3.9 CI not reported | A) Non-inferiority proven  B) Inconclusive |
| Cao et al.  2017  [132] | 2009-2010  Multicenter  Adults | Double blinded | Pelvic inflammatory disease | Morinidazole IV with Aztreonam IV or Etimicin IV (N=159/168)  Orinidazole IV with Aztreonam IV or Etimicin IV (N=153/170) | Clinical response  NIM = 10%  ITT: 5.8 95% CI -4.1 to 5.1  PP: 0.07 95% CI -3.8 to 4 | A) Non-inferiority proven  B) Non-inferiority proven |
| Koksal et al.  2013  [133] | 2011  Single center  ? | Open label | H pylori infection | Quadruple therapy BID regimen (N=45)  Quadruple therapy QID regimen (N=45) | Eradication  NIM = 10%  ITT: -15.6 CI not reported  PP: -3.3 CI not reported | A) Non-inferiority proven  B) Inconclusive |
| Molton et al.  2019  [134] | 2013-2017  Multicenter  Adults | Open label | Klebsiella liver abscess | Ciprofloxacin PO (N=59/74)  Ceftriaxone IV (N=68/78) | Clinical cure at 12 weeks  NIM = 12%  ITT: 3.6 95% CI -4.9 to 12.8  PP: 1.2 95% CI -6.4 to 9 | A) Non-inferiority proven  B) Non-inferiority proven |
| Cranendonk et al.  2019  [135] | 2014-2017  Multicenter  Adults | Double blinded | Severe cellulitis | 6 days of Cloxacillin IV or PO (N=66/73)  12 days of Cloxacillin IV or PO (N=66/76) | Clinical cure at day 14 without relapse  NIM = 10%  ITT: 0.7 95% C -15 to 16.3  PP: -7.6 95% CI -23.8 to 9.2 | A) Inconclusive  B) Inconclusive  Stopped early due to slow recruitment and lack of funding |
| File et al.  2019  [136] | 2016-2017  Multicenter  Adults | Double blinded | Community acquired pneumonia | Lefamulin IV then PO (N=236/273)  Moxifloxacin IV then PO (N=245/273) | Clinical response  NIM = 10%  mITT: -2.6 95% CI -8.9 to 3.9  PP: -2.5 95% CI -8.4 to 3.4 | A) Non-inferiority proven  B) Non-inferiority proven |
| Clarke et al.  2019  [137] | 2012-2016  Multicenter  Adults | Open label | Lower limb erysipelas or cellulitis | 24 hours of IV then PO antibiotics (N=38/39)  >=72 hrs of IV antibiotics (N=39/41) | Clinical resolution  NIM = 10%  ITT: -5.8 95% CI -22.5 to 10.7  PP: -8.2 95% CI -23.7 to 7.4 | A) Inconclusive  B) Inconclusive  Stopped early due to slow recruitment |
| Yahav et al.  2019  [138] | 2013-2017  Multicenter  Adults | Open label | Gram negative bacteremia | 7 days of antibiotic (N=280/306)  14 days of antibiotic (N=276/298) | Composite outcome of mortality, complications, readmissions, prolonged hospital stay  NIM = 10%  ITT: 2.6 95% CI -5.3 to 10.5  PP: 2.1 95% CI -6.2 to 10.4 | A) Non-inferiority proven  B) Non-inferiority proven |
| Muntenu et al.  2017  [139] | 2012-2015  Single center  Adults | Open label | H pylori infection | 10 days of sequential therapy (N=50)  14 days of quadruple regimen (N=51) | Eradication  NIM = 15%  mITT: 6.4 95% CI -14 to 27  PP: -10 95% CI not reported | A) Non-inferiority proven  B) Non-inferiority proven  Stopped early due to slow recruitment |
| Tancawan et al.  2015  [140] | ?  Multicenter  Adults | Single blinded | Odontogenic infection | Amoxicillin-Clavunate PO (N=223/234)  Clindamycin PO (N=229/237) | Clinical success  NIM = 10%  ITT: -2.3 95% CI -9 to 4.3  PP: -1.5 95% CI -7.7 to 4.7 | A) Non-inferiority proven  B) Non-inferiority proven |
| Fomin et al.  2005  [141] | 2002-2004  Multicenter  Adults | Double blinded | Complicated intra-abdominal infection | TIgecycline IV (N=265/322)  Imipenem-cilastatin IV (N=258/319) | Clinical cure  NIM = 15%  mITT: 2 95% CI -3.7 to 7.5  PP: 1.4 95% CI -4 to 6.8 | A) Non-inferiority proven  B) Non-inferiority proven |
| O’Riordan et al.  2019  [142] | 2015-2016  Multicenter  Adults | Double blinded | Acute bacterial skin and skin structure infection | Omadacycline IV then PO (N=298/316)  Linezolid IV then PO (N=294/311) | Early clinical response  NIM = 10%  mITT: -0.7 95% CI -6.3 to 4.9  PP: -1.9 95% CI: -6.1 to 2.1 | A) Non-inferiority proven  B) Non-inferiority proven |
| Graham et al.  2002  [143] | 1998-1999  Multicenter  Adults | Double blinded | Complicated skin and skin structure infection | Ertapenem IV (N=185/274)  Piperacillin-Tazobactam IV (N=174/266) | Clinical response  NIM = 15%  mITT: -3.6 95% CI -11.6 to 4.4  PP: -2 95% CI -10.2 to 6.2 | A) Non-inferiority proven  B) Non-inferiority proven |
| Lipsky et al.  2005  [144] | 2001-2004  Multicenter  Adults | Double blinded | Diabetic foot infection | Ertapenem IV +/- Vancomycin IV, then step down to Amoxicillin-Clavunate PO (N=226/289)  Piperacillin-Tazobactam IV +/- Vancomycin IV, then step down to Amoxicillin-Clavunate PO (N=219/285) | Favorable clinical response  NIM = 15%  mITT: 5 95% CI -2.6 to 12.5  PP: 1.9 95% CI -2.9 to 6.9 | A) Non-inferiority proven  B) Non-inferiority proven |
| Ortiz-Ruiz et al.  2002  [145] | 1998-1999  Multicenter  Adults | Double blinded | Community acquired pneumonia | Ertapenem IV then step down to Amoxicillin-Clavunate PO (N=244)  Ceftriaxone IV then step down to Amoxicillin-Clavunate PO (N=258) | Favorable clinical response  NIM = 10%  mITT: 0.1 CI not reported  PP: 1 95% CI -4.9 to 7 | A) Non-inferiority proven  B) Non-inferiority proven |
| Vetter et al.  2002  [146] | 1998-2000  Multicenter  Adults | Double blinded | Community acquired pneumonia | Ertapenem IV then step down to Amoxicillin-Clavunate PO (N=239)  Ceftriaxone IV then step down to Amoxicillin-Clavunate PO (N=125) | Clinical response  NIM = 10%  mITT: -0.5 CI not reported  PP: -1.5 95% CI -8.6 to 5.7 | A) Non-inferiority proven  B) Non-inferiority proven |
| Jimenez-Cruz et al.  2002  [147] | 1998-2000  Multicenter  Adults | Double blinded | Complicated urinary tract infection | Ertapenem IV then step down to Ciprofloxacin PO (N=97/131)  Ceftriaxone IV then step down to Ciprofloxacin PO (N=53/71) | Microbiologic eradication  NIM = 20%  mITT: 4.3 95% CI -7.5 to 16.1  PP: 0.6 95% CI -12.9 to 14.1 | A) Non-inferiority proven  B) Non-inferiority proven |
| Tomera et al.  2002  [148] | 1998-2000  Multicenter  Adults | Double blinded | Complicated urinary tract infection | Ertapenem IV then step down to Ciprofloxacin PO (N=159/219)  Ceftriaxone IV then step down to Ciprofloxacin PO (N=171/242) | Microbiologic eradication  NIM = 10%  mITT: 1.4 95% CI -4 to 6.9  PP: -1.2 95% CI -7.6 to 5.1 | A) Non-inferiority proven  B) Non-inferiority proven |
| Roy et al.  2003  [149] | 1998-2000  Multicenter  Adults | Double blinded | Acute pelvic infection | Ertapenem IV (N=163/211)  Piperacillin-Tazobactam IV (N=153/191) | Clinical response  NIM = 10%  mITT: -2.1 95% CI -9.2 to 5  PP: 2.4 95% CI -4 to 8.8 | A) Non-inferiority proven  B) Non-inferiority proven |
| Tellier et al.  2004  [150] | 1999-2001  Multicenter  Adults | Double blinded | Community acquired pneumonia | Telithromycin 800mg po daily for 5 days (N=287)  Telithromycin 800mg po daily for 7 days (N=191)  Clarithromycin 500mg po bid for 10 days (N=181) | Clinical cure  NIM = 15%  Telithromycin for 5 days vs. Clarithromycin:  mITT: 1.2 CI not reported  PP: -2.5 95% CI -9.7 to 4.7  Telithromycin for 7 days vs. Clarithromycin:  mITT: 1 CI not reported  PP; -2.5 95% CI -9.7 to 4.7 | A) Non-inferiority proven  B) Non-inferiority proven |
| Dunbar et al.  2004  [151] | 1998-1999  Multicenter  Adults | Double blinded | Community acquired pneumonia | Telithromycin po for 10 days (N=162/204)  Clarithromycin po for 10 days (N=156/212) | Clinical cure  NIM = 15%  mITT: -1.7 95% CI -9.9 to 6.5  PP: -0.2 95% CI -7.8 to 7.5 | A) Non-inferiority proven  B) Non-inferiority proven |
| Stryjewski et al.  2008  [152] | 2005-2006  Multicenter  Adults | Double blinded | Complicated skin and skin structure infections caused by gram positive organisms | Telavancin IV +/- Aztreonam IV +/- Metronidazole IV (N=745/928)  Vancomycin IV +/- Aztreonam IV +/- Metronidazole IV (N=744/939) | Clinical response  NIM = 10%  ITT: 2.3 95% CI -1.6 to 6.2  PP: 1.2 95% CI -2.1 to 4.6 | A) Non-inferiority proven  B) Non-inferiority proven |
| Naber et al.  2009  [153] | 2003-2006  Multicenter  Adults | Double blinded | Complicated urinary tract infection and pyelonephritis | Doripenem IV then step down to Levofloxacin PO (N=280/327)  Levofloxacin IV then PO (N=265/321) | Microbiologic cure  NIM = 10%  mITT: 1 95% CI -5.6 to 7.6  PP: -1.3 95% CI -8 to 5.5 | A) Non-inferiority proven  B) Non-inferiority proven |
| Breedt et al.  2005  [154] | 2002-2003  Multicenter  Adults | Double blinded | Complicated skin and skin structure infection | Tigecycline IV (N=223/261)  Vancomycin IV and Aztreonam IV (N=213/259) | Clinical response  NIM = 15%  mITT: -2.6 95% CI -9 to 3.8  PP: -4.7 95% CI -10.2 to 0.8 | A) Non-inferiority proven  B) Non-inferiority proven |

CI = confidence interval, ITT = intention-to-treat analysis, mITT = modified intention-to-treat analysis, NIM = non-inferiority margin, PP = per-protocol analysis

^a^The point estimate and confidence interval may have been switched in terms of positive and negative so that all of the point estimates and confidence align such that <0 represents new treatment worse and >0 represents new treatment better

**Appendix Table 2. Risk of bias assessment for each individual study**

| 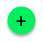 | Low risk |
| --- | --- |
| 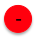 | High risk |
| 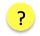 | Unclear |

|  | Randomization sequence generation | Allocation concealment | Performance bias | Detection bias | Attrition bias | Reporting bias |
| --- | --- | --- | --- | --- | --- | --- |
| Nieuwkoop et al. 2017 [1] | 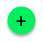 | 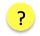 | 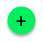 | 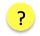 | 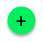 | 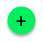 |
| Chosidow et al. 2005 [2] | 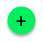 | 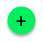 | 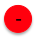 | 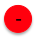 | 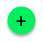 | 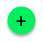 |
| Harbarth et al. 2015 [3] | 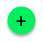 | 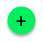 | 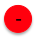 | 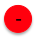 | 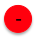 | 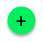 |
| Brack et al. 2012 [4] | 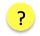 | 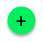 | 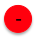 | 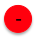 | 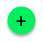 | 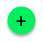 |
| Goyal et al. 2018 [5] | 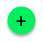 | 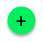 | 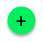 | 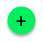 | 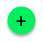 | 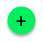 |
| Bernard et al. 2002 [6] | 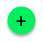 | 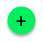 | 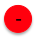 | 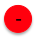 | 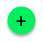 | 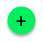 |
| Liu et al. 2019 [7] | 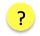 | 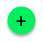 | 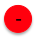 | 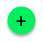 | 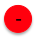 | 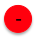 |
| Rudrabhatla et al. 2018 [8] | 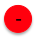 | 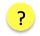 | 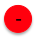 | 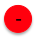 | 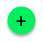 | 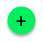 |
| Bernard et al. 2015 [9] | 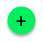 | 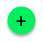 | 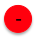 | 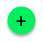 | 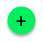 | 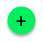 |
| Cornely et al. 2012 [10] | 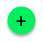 | 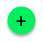 | 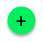 | 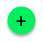 | 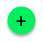 | 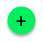 |
| Mitja et al. 2012 [11] | 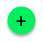 | 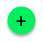 | 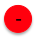 | 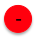 | 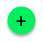 | 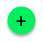 |
| Mikamo et al. 2018 [12] | 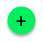 | 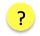 | 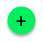 | 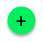 | 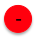 | 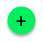 |
| Snyman et al. 2009 [13] | 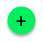 | 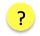 | 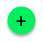 | 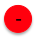 | 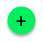 | 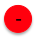 |
| Malfertheiner et al. 2011 [14] | 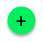 | 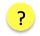 | 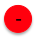 | 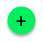 | 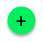 | 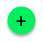 |
| Barrera et al. 2016 [15] | 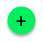 | 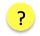 | 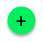 | 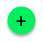 | 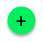 | 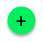 |
| Dalen et al. 2018 [16] | 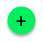 | 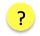 | 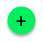 | 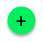 | 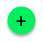 | 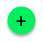 |
| Bowen et al. 2014 [17] | 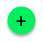 |  |  |  |  |  |
| Chen et al. 2016 [18] |  |  |  |  |  |  |
| Zhong et al. 2015 [19] |  |  |  |  |  |  |
| Stahlgren et al. 2019 [20] |  |  |  |  |  |  |
| Chandra et al. 2008 [21] |  |  |  |  |  |  |
| Pullman et al. 2017 [22] |  |  |  |  |  |  |
| Wagenlehner et al. 2015 [23] |  |  |  |  |  |  |
| Gerding et al. 2019 [24] |  |  |  |  |  |  |
| Paul et al. 2015 [25] |  |  |  |  |  |  |
| Nathan et al. 2005 [26] |  |  |  |  |  |  |
| Rhee et al. 2015 [27] |  |  |  |  |  |  |
| Llor et al. 2017 [28] |  |  |  |  |  |  |
| Xu et al. 2016 [29] |  |  |  |  |  |  |
| Dryden et al. 2016 [30] |  |  |  |  |  |  |
| Ren et al. 2017 [31] |  |  |  |  |  |  |
| Heystek et al. 2009 [32] |  |  |  |  |  |  |
| Bocquet et al. 2012 [33] |  |  |  |  |  |  |
| Bartacek et al. 2009 [34] |  |  |  |  |  |  |
| Torres et al. 2018 [35] |  |  |  |  |  |  |
| Gyssens et al. 2011 [36} |  |  |  |  |  |  |
| Zhao et al. 2016 [37] |  |  |  |  |  |  |
| Tanaseanu et al. 2009 [38] |  |  |  |  |  |  |
| Towfigh et al. 2010 [39] |  |  |  |  |  |  |
| Yakovlev et al. 2006 [40] |  |  |  |  |  |  |
| Nakane et al. 2015 [41] |  |  |  |  |  |  |
| Waele et al. 2013 [42] |  |  |  |  |  |  |
| Namias et al. 2007 [43] |  |  |  |  |  |  |
| Lora-Tamayo et al. 2016 [44] |  |  |  |  |  |  |
| Ibrahim et al. 2019 [45] |  |  |  |  |  |  |
| Solomkin et al. 2009 [46] |  |  |  |  |  |  |
| Lv et al. 2017 [47] |  |  |  |  |  |  |
| Yuan et al. 2018 [48] |  |  |  |  |  |  |
| Liou et al. 2018 [49] |  |  |  |  |  |  |
| Aseffa et al. 2016 [50] |  |  |  |  |  |  |
| File et al. 2001 [51] |  |  |  |  |  |  |
| Harris et al. 2018 [52] |  |  |  |  |  |  |
| Aliberti et al. 2017 [53] |  |  |  |  |  |  |
| GIusti et al. 2016 [54] |  |  |  |  |  |  |
| Oliva et al. 2005 [55] |  |  |  |  |  |  |
| Judlin et al. 2010 [56] |  |  |  |  |  |  |
| Zhang et al. 2015 [57] |  |  |  |  |  |  |
| Ross et al. 2006 [58] |  |  |  |  |  |  |
| Nicholson et al. 2012 [59] |  |  |  |  |  |  |
| Wilson et al. 2012 [60] |  |  |  |  |  |  |
| Rob et al. 2019 [61] |  |  |  |  |  |  |
| Petitpretz et al. 2007 [62] |  |  |  |  |  |  |
| Moussaoui et al. 2006 [63] |  |  |  |  |  |  |
| Fabian et al. 2005 [64] |  |  |  |  |  |  |
| Siquier et al. 2006 [65] |  |  |  |  |  |  |
| Corey et al. 2010 [66] |  |  |  |  |  |  |
| Wilcox et al. 2010 [67] |  |  |  |  |  |  |
| File et al. 2007 [68] |  |  |  |  |  |  |
| File et al. 2011 [69] |  |  |  |  |  |  |
| Low et al. 2011 [70] |  |  |  |  |  |  |
| Huang et al. 2018 [71] |  |  |  |  |  |  |
| Corey et al. 2014 [72] |  |  |  |  |  |  |
| Stets et al. 2019 [73] |  |  |  |  |  |  |
| O’Riordan et al. 2019 [74] |  |  |  |  |  |  |
| Federico et al. 2012 [75] |  |  |  |  |  |  |
| Freire et al. 2010 [76] |  |  |  |  |  |  |
| Lauf et al. 2014 [77] |  |  |  |  |  |  |
| Dartois et al. 2008 [78] |  |  |  |  |  |  |
| Bergallo et al. 2009 [79] |  |  |  |  |  |  |
| Sacchidanad et al. 2005 [80] |  |  |  |  |  |  |
| Louie et al. 2011 [81] |  |  |  |  |  |  |
| Solomkin et al. 2015 [82] |  |  |  |  |  |  |
| Weiss et al. 2009 [83] |  |  |  |  |  |  |
| Molina-Infante et al. 2013 [84] |  |  |  |  |  |  |
| Mazuski et al. 2016 [85] |  |  |  |  |  |  |
| Chung et al. 2011 [86] |  |  |  |  |  |  |
| Dore et al. 2011 [87] |  |  |  |  |  |  |
| Tsay et al. 2015 [88] |  |  |  |  |  |  |
| Bohbot et al. 2010 [89] |  |  |  |  |  |  |
| Jindani et al. 2014 [90] |  |  |  |  |  |  |
| Lienhardt et al. 2011 [91] |  |  |  |  |  |  |
| Merle et al. 2014 [92] |  |  |  |  |  |  |
| Gillespie et al. 2014 [93] |  |  |  |  |  |  |
| Nunn et al. 2019 [94] |  |  |  |  |  |  |
| Kaye et al. 2018 [95] |  |  |  |  |  |  |
| File et al. 2004 [96] |  |  |  |  |  |  |
| Van Rensburg et al. 2010 [97] |  |  |  |  |  |  |
| Desrosiers et al. 2008 [98] |  |  |  |  |  |  |
| Lucasti et al. 2008 [99] |  |  |  |  |  |  |
| Corey et al. 2015 [100] |  |  |  |  |  |  |
| Clegg et al. 2006 [101] |  |  |  |  |  |  |
| Zanetti et al. 2003 [102] |  |  |  |  |  |  |
| Rimoin et al. 2011 [103] |  |  |  |  |  |  |
| Uranga et al. 2016 [104] |  |  |  |  |  |  |
| Li et al. 2019 [105] |  |  |  |  |  |  |
| Naber et al. 2004 [106] |  |  |  |  |  |  |
| Noel et al. 2008 [107] |  |  |  |  |  |  |
| UpChurch et al. 2006 [108] |  |  |  |  |  |  |
| O’Riordan et al. 2018 [109] |  |  |  |  |  |  |
| Chastre et al. 2008 [110] |  |  |  |  |  |  |
| Leroy et al. 2005 [111] |  |  |  |  |  |  |
| Torres et al. 2008 [112] |  |  |  |  |  |  |
| Agweyu et al. 2015 [113] |  |  |  |  |  |  |
| Lojanapiwat et al. 2019 [114] |  |  |  |  |  |  |
| Iversen et al. 2019 [115] |  |  |  |  |  |  |
| Solomkin et al. 2017 [116] |  |  |  |  |  |  |
| Awad et al. 2014 [117] |  |  |  |  |  |  |
| Bradley et al. 2007 [118] |  |  |  |  |  |  |
| Klausner et al. 2007 [119] |  |  |  |  |  |  |
| Fowler et al. 2006 [120] |  |  |  |  |  |  |
| Saar et al. 2019 [121] |  |  |  |  |  |  |
| Fourcroy et al. 2005 [122] |  |  |  |  |  |  |
| Hooton et al. 2012 [123] |  |  |  |  |  |  |
| Noel et al. 2008 [124] |  |  |  |  |  |  |
| Peterson et al. 2008 [125] |  |  |  |  |  |  |
| English et al. 2012 [126] |  |  |  |  |  |  |
| Rubinstein et al. 2011 [127] |  |  |  |  |  |  |
| Mir et al. 2019 [128] |  |  |  |  |  |  |
| Solomkin et al. 2019 [129] |  |  |  |  |  |  |
| Chen et al. 2018 [130] |  |  |  |  |  |  |
| Sapmaz et al. 2017 [131] |  |  |  |  |  |  |
| Cao et al. 2017 [132] |  |  |  |  |  |  |
| Koksal et al. 2013 [133] |  |  |  |  |  |  |
| Molton et al. 2019 [134] |  |  |  |  |  |  |
| Cranendonk et al. 2019 [135] |  |  |  |  |  |  |
| File et al. 2019 [136] |  |  |  |  |  |  |
| Clarke et al. 2019 [137] |  |  |  |  |  |  |
| Yahav et al. 2019 [138] |  |  |  |  |  |  |
| Muntenu et al. 2017 [139] |  |  |  |  |  |  |
| Tancawan et al. 2015 [140] |  |  |  |  |  |  |
| Fomin et al. 2005 [141] |  |  |  |  |  |  |
| O’Riordan et al. 2019 [142] |  |  |  |  |  |  |
| Graham et al. 2002 [143] |  |  |  |  |  |  |
| Lipsky et al. 2005 [144] |  |  |  |  |  |  |
| Ortiz-Ruiz et al. 2002 [145] |  |  |  |  |  |  |
| Vetter et al. 2002 [146] |  |  |  |  |  |  |
| Jimenez-Cruz et al. 2002 [147] |  |  |  |  |  |  |
| Tomera et al. 2002 [148] |  |  |  |  |  |  |
| Roy et al. 2003 [149] |  |  |  |  |  |  |
| Tellier et al. 2004 [150] |  |  |  |  |  |  |
| Dunbar et al. 2004 [151] |  |  |  |  |  |  |
| Stryjewski et al. 2008 [152] |  |  |  |  |  |  |
| Naber et al. 2009 [153] |  |  |  |  |  |  |
| Breedt et al. 2005 [154] |  |  |  |  |  |  |

**References**

1. van Nieuwkoop C, van der Starre WE, Stalenhoef JE, van Aartrijk AM, van der Reijden TJ, Vollaard AM, Delfos NM, van’t Wout JW, Blom JW, Spelt IC, Leyten EM. Treatment duration of febrile urinary tract infection: a pragmatic randomized, double-blind, placebo-controlled non-inferiority trial in men and women. BMC medicine. 2017 Dec 1;15(1):70.

2. Chosidow O, Bernard P, Berbis P, Humbert P, Crickx B, Jarlier V. Cloxacillin versus pristinamycin for superficial pyodermas: a randomized, open-label, non-inferiority study. Dermatology. 2005;210(4):370-4.

3. Harbarth S, Von Dach E, Pagani L, Macedo-Vinas M, Huttner B, Olearo F, Emonet S, Uçkay I. Randomized non-inferiority trial to compare trimethoprim/sulfamethoxazole plus rifampicin versus linezolid for the treatment of MRSA infection. Journal of Antimicrobial Chemotherapy. 2015 Jan 1;70(1):264-72.

4. Brack E, Bodmer N, Simon A, Leibundgut K, Kühne T, Niggli FK, Ammann RA. First‐day step‐down to oral outpatient treatment versus continued standard treatment in children with cancer and low‐risk fever in neutropenia. A randomized controlled trial within the multicenter SPOG 2003 FN study. Pediatric blood & cancer. 2012 Sep;59(3):423-30.

5. Goyal V, Grimwood K, Byrnes CA, Morris PS, Masters IB, Ware RS, McCallum GB, Binks MJ, Marchant JM, Van Asperen P, O'Grady KA. Amoxicillin–clavulanate versus azithromycin for respiratory exacerbations in children with bronchiectasis (BEST-2): a multicentre, double-blind, non-inferiority, randomised controlled trial. The Lancet. 2018 Oct 6;392(10154):1197-206.

6. Bernard P, Chosidow O, Vaillant L. Oral pristinamycin versus standard penicillin regimen to treat erysipelas in adults: randomised, non-inferiority, open trial. Bmj. 2002 Oct 19;325(7369):864.

7. Liu JW, Chen YH, Lee WS, Lin JC, Huang CT, Lin HH, Liu YC, Chuang YC, Tang HJ, Chen YS, Ko WC. Randomized noninferiority trial of cefoperazone-sulbactam versus cefepime in the treatment of hospital-acquired and healthcare-associated pneumonia. Antimicrobial agents and chemotherapy. 2019 Aug 1;63(8):e00023-19.

8. Rudrabhatla P, Deepanjali S, Mandal J, Swaminathan RP, Kadhiravan T. Stopping the effective non-fluoroquinolone antibiotics at day 7 vs continuing until day 14 in adults with acute pyelonephritis requiring hospitalization: A randomized non-inferiority trial. PloS one. 2018;13(5).

9. Bernard L, Dinh A, Ghout I, Simo D, Zeller V, Issartel B, Le Moing V, Belmatoug N, Lesprit P, Bru JP, Therby A. Antibiotic treatment for 6 weeks versus 12 weeks in patients with pyogenic vertebral osteomyelitis: an open-label, non-inferiority, randomised, controlled trial. The Lancet. 2015 Mar 7;385(9971):875-82.

10. Cornely OA, Crook DW, Esposito R, Poirier A, Somero MS, Weiss K, Sears P, Gorbach S, OPT-80-004 Clinical Study Group. Fidaxomicin versus vancomycin for infection with Clostridium difficile in Europe, Canada, and the USA: a double-blind, non-inferiority, randomised controlled trial. The Lancet infectious diseases. 2012 Apr 1;12(4):281-9.

11. Mitjà O, Hays R, Ipai A, Penias M, Paru R, Fagaho D, de Lazzari E, Bassat Q. Single-dose azithromycin versus benzathine benzylpenicillin for treatment of yaws in children in Papua New Guinea: an open-label, non-inferiority, randomised trial. The Lancet. 2012 Jan 28;379(9813):342-7.

12. Mikamo H, Tateda K, Yanagihara K, Kusachi S, Takesue Y, Miki T, Oizumi Y, Gamo K, Hashimoto A, Toyoshima J, Kato K. Efficacy and safety of fidaxomicin for the treatment of Clostridioides (Clostridium) difficile infection in a randomized, double-blind, comparative phase III study in Japan. Journal of infection and chemotherapy. 2018 Sep 1;24(9):744-52.

13. Snyman JR, Schoeman HS, Grobusch MP, Henning M, Rabie W, Hira M, Parshotam K, Mithal Y, Singh S, Ramdas Z. Generic versus non-generic formulation of extended-release clarithromycin in patients with community-acquired respiratory tract infections. Clinical drug investigation. 2009 Apr 1;29(4):265-74.

14. Malfertheiner P, Bazzoli F, Delchier JC, Celiñski K, Giguère M, Rivière M, Mégraud F, Pylera Study Group. Helicobacter pylori eradication with a capsule containing bismuth subcitrate potassium, metronidazole, and tetracycline given with omeprazole versus clarithromycin-based triple therapy: a randomised, open-label, non-inferiority, phase 3 trial. The Lancet. 2011 Mar 12;377(9769):905-13.

15. Barrera CM, Mykietiuk A, Metev H, Nitu MF, Karimjee N, Doreski PA, Mitha I, Tanaseanu CM, Molina JM, Antonovsky Y, Van Rensburg DJ. Efficacy and safety of oral solithromycin versus oral moxifloxacin for treatment of community-acquired bacterial pneumonia: a global, double-blind, multicentre, randomised, active-controlled, non-inferiority trial (SOLITAIRE-ORAL). The Lancet Infectious Diseases. 2016 Apr 1;16(4):421-30.

16. Dalen D, Fry A, Campbell SG, Eppler J, Zed PJ. Intravenous cefazolin plus oral probenecid versus oral cephalexin for the treatment of skin and soft tissue infections: a double-blind, non-inferiority, randomised controlled trial. Emergency Medicine Journal. 2018 Aug 1;35(8):492-8.

17. Bowen AC, Tong SY, Andrews RM, O'Meara IM, McDonald MI, Chatfield MD, Currie BJ, Carapetis JR. Short-course oral co-trimoxazole versus intramuscular benzathine benzylpenicillin for impetigo in a highly endemic region: an open-label, randomised, controlled, non-inferiority trial. The Lancet. 2014 Dec 13;384(9960):2132-40.

18. Chen Q, Zhang W, Fu Q, Liang X, Liu W, Xiao S, Lu H. Rescue therapy for Helicobacter pylori eradication: a randomized non-inferiority trial of amoxicillin or tetracycline in bismuth quadruple therapy. American Journal of Gastroenterology. 2016 Dec 1;111(12):1736-42.

19. Zhong NS, Sun T, Zhuo C, D'Souza G, Lee SH, Lan NH, Chiang CH, Wilson D, Sun F, Iaconis J, Melnick D. Ceftaroline fosamil versus ceftriaxone for the treatment of Asian patients with community-acquired pneumonia: a randomised, controlled, double-blind, phase 3, non-inferiority with nested superiority trial. The Lancet Infectious Diseases. 2015 Feb 1;15(2):161-71.

20. Ståhlgren GS, Tyrstrup M, Edlund C, Giske CG, Mölstad S, Norman C, Rystedt K, Sundvall PD, Hedin K. Penicillin V four times daily for five days versus three times daily for 10 days in patients with pharyngotonsillitis caused by group A streptococci: randomised controlled, open label, non-inferiority study. bmj. 2019 Oct 4;367:l5337.

21. MASCOT Study Group, Chandra A, Dhar P, Dharap S, Goel A, Gupta R, Hardikar JV, Kapoor VK, Mathur AK, Modi P, Narwaria M. Cefoperazone-sulbactam for treatment of intra-abdominal infections: results from a randomized, parallel group study in India. Surgical infections. 2008 Jun 1;9(3):367-76.

22. Pullman J, Gardovskis J, Farley B, Sun E, Quintas M, Lawrence L, Ling R, Cammarata S, PROCEED Study Group. Efficacy and safety of delafloxacin compared with vancomycin plus aztreonam for acute bacterial skin and skin structure infections: a phase 3, double-blind, randomized study. Journal of Antimicrobial Chemotherapy. 2017 Dec 1;72(12):3471-80.

23. Wagenlehner FM, Umeh O, Steenbergen J, Yuan G, Darouiche RO. Ceftolozane-tazobactam compared with levofloxacin in the treatment of complicated urinary-tract infections, including pyelonephritis: a randomised, double-blind, phase 3 trial (ASPECT-cUTI). The Lancet. 2015 May 16;385(9981):1949-56.

24. Gerding DN, Cornely OA, Grill S, Kracker H, Marrast AC, Nord CE, Talbot GH, Buitrago M, Diaconescu IG, de Oliveira CM, Preotescu L. Cadazolid for the treatment of Clostridium difficile infection: results of two double-blind, placebo-controlled, non-inferiority, randomised phase 3 trials. The Lancet Infectious Diseases. 2019 Mar 1;19(3):265-74.

25. Paul M, Bishara J, Yahav D, Goldberg E, Neuberger A, Ghanem-Zoubi N, Dickstein Y, Nseir W, Dan M, Leibovici L. Trimethoprim-sulfamethoxazole versus vancomycin for severe infections caused by meticillin resistant Staphylococcus aureus: randomised controlled trial. bmj. 2015 May 14;350:h2219.

26. Nathan N, Borel T, Djibo A, Evans D, Djibo S, Corty JF, Guillerm M, Alberti KP, Pinoges L, Guerin PJ, Legros D. Ceftriaxone as effective as long-acting chloramphenicol in short-course treatment of meningococcal meningitis during epidemics: a randomised non-inferiority study. The Lancet. 2005 Jul 23;366(9482):308-13.

27. Rhee CK, Chang JH, gene Choi E, kuk Kim H, Kwon YS, Kyung SY, Lee JH, Park MJ, Yoo KH, Oh YM. Zabofloxacin versus moxifloxacin in patients with COPD exacerbation: a multicenter, double-blind, double-dummy, randomized, controlled, phase III, non-inferiority trial. International journal of chronic obstructive pulmonary disease. 2015;10:2265.

28. Llor C, Pérez A, Carandell E, García-Sangenís A, Rezola J, Llorente M, Gestoso S, Bobé F, Román-Rodríguez M, Cots JM, Hernández S. Efficacy of high doses of penicillin versus amoxicillin in the treatment of uncomplicated community acquired pneumonia in adults. A non-inferiority controlled clinical trial. Atencion primaria. 2019 Jan 1;51(1):32-9.

29. Xu ZR, Ran XW, Xian Y, Yan XD, Yuan GY, Mu SM, Shen JF, Zhang BS, Gan WJ, Wang J. Ertapenem versus piperacillin/tazobactam for diabetic foot infections in China: a phase 3, multicentre, randomized, double-blind, active-controlled, non-inferiority trial. Journal of Antimicrobial Chemotherapy. 2016 Jun 1;71(6):1688-96.

30. Dryden M, Zhang Y, Wilson D, Iaconis JP, Gonzalez J. A Phase III, randomized, controlled, non-inferiority trial of ceftaroline fosamil 600 mg every 8 h versus vancomycin plus aztreonam in patients with complicated skin and soft tissue infection with systemic inflammatory response or underlying comorbidities. Journal of Antimicrobial Chemotherapy. 2016 Dec 1;71(12):3575-84.

31. Ren H, Li X, Ni ZH, Niu JY, Cao B, Xu J, Cheng H, Tu XW, Ren AM, Hu Y, Xing CY. Treatment of complicated urinary tract infection and acute pyelonephritis by short-course intravenous levofloxacin (750 mg/day) or conventional intravenous/oral levofloxacin (500 mg/day): prospective, open-label, randomized, controlled, multicenter, non-inferiority clinical trial. International urology and nephrology. 2017 Mar 1;49(3):499-507.

32. Heystek M, Ross JD. A randomized double-blind comparison of moxifloxacin and doxycycline/metronidazole/ciprofloxacin in the treatment of acute, uncomplicated pelvic inflammatory disease. International journal of STD & AIDS. 2009 Oct;20(10):690-5.

33. Bocquet N, Alaoui AS, Jais JP, Gajdos V, Guigonis V, Lacour B, Chéron G. Randomized trial of oral versus sequential IV/oral antibiotic for acute pyelonephritis in children. Pediatrics. 2012 Feb 1;129(2):e269-75.

34. Bartacek A, Schütt D, Panosch B, Borek M, Rimstar® 4-FDC Study Group. Comparison of a four-drug fixed-dose combination regimen with a single tablet regimen in smear-positive pulmonary tuberculosis. The International journal of tuberculosis and lung disease. 2009 Jun 1;13(6):760-6.

35. Torres A, Zhong N, Pachl J, Timsit JF, Kollef M, Chen Z, Song J, Taylor D, Laud PJ, Stone GG, Chow JW. Ceftazidime-avibactam versus meropenem in nosocomial pneumonia, including ventilator-associated pneumonia (REPROVE): a randomised, double-blind, phase 3 non-inferiority trial. The Lancet Infectious Diseases. 2018 Mar 1;18(3):285-95.

36. Gyssens IC, Dryden M, Kujath P, Nathwani D, Schaper N, Hampel B, Reimnitz P, Alder J, Arvis P. A randomized trial of the efficacy and safety of sequential intravenous/oral moxifloxacin monotherapy versus intravenous piperacillin/tazobactam followed by oral amoxicillin/clavulanate for complicated skin and skin structure infections. Journal of antimicrobial chemotherapy. 2011 Nov 1;66(11):2632-42.

37. Zhao T, Chen LA, Wang P, Tian G, Ye F, Zhu H, He B, Zhang B, Shao C, Jie Z, Gao X. A randomized, open, multicenter clinical study on the short course of intravenous infusion of 750 mg of levofloxacin and the sequential standard course of intravenous infusion/oral administration of 500 mg of levofloxacin for treatment of community-acquired pneumonia. Journal of thoracic disease. 2016 Sep;8(9):2473.

38. Tanaseanu C, Milutinovic S, Calistru PI, Strausz J, Zolubas M, Chernyak V, Dartois N, Castaing N, Gandjini H, Cooper CA, 313 Study Group. Efficacy and safety of tigecycline versus levofloxacin for community-acquired pneumonia. BMC pulmonary medicine. 2009 Dec 1;9(1):44.

39. Towfigh S, Pasternak J, Poirier A, Leister H, Babinchak T. A multicentre, open-label, randomized comparative study of tigecycline versus ceftriaxone sodium plus metronidazole for the treatment of hospitalized subjects with complicated intra-abdominal infections. Clinical microbiology and infection. 2010 Aug 1;16(8):1274-81.

40. Yakovlev SV, Stratchounski LS, Woods GL, Adeyi B, McCarroll KA, Ginanni JA, Friedland IR, Wood CA, DiNubile MJ. Ertapenem versus cefepime for initial empirical treatment of pneumonia acquired in skilled-care facilities or in hospitals outside the intensive care unit. European Journal of Clinical Microbiology and Infectious Diseases. 2006 Oct 1;25(10):633.

41. Nakane T, Tamura K, Hino M, Tamaki T, Yoshida I, Fukushima T, Tatsumi Y, Nakagawa Y, Hatanaka K, Takahashi T, Akiyama N. Cefozopran, meropenem, or imipenem–cilastatin compared with cefepime as empirical therapy in febrile neutropenic adult patients: A multicenter prospective randomized trial. Journal of Infection and Chemotherapy. 2015 Jan 1;21(1):16-22.

42. De Waele JJ, Tellado JM, Alder J, Reimnitz P, Jensen M, Hampel B, Arvis P. Randomised clinical trial of moxifloxacin versus ertapenem in complicated intra-abdominal infections: results of the PROMISE study. International journal of antimicrobial agents. 2013 Jan 1;41(1):57-64.

43. Namias N, Solomkin JS, Jensen EH, Tomassini JE, Abramson MA. Randomized, multicenter, double-blind study of efficacy, safety, and tolerability of intravenous ertapenem versus piperacillin/tazobactam in treatment of complicated intra-abdominal infections in hospitalized adults. Surgical infections. 2007 Feb 1;8(1):15-28.

44. Lora-Tamayo J, Euba G, Cobo J, Horcajada JP, Soriano A, Sandoval E, Pigrau C, Benito N, Falgueras L, Palomino J, del Toro MD. Short-versus long-duration levofloxacin plus rifampicin for acute staphylococcal prosthetic joint infection managed with implant retention: a randomised clinical trial. International journal of antimicrobial agents. 2016 Sep 1;48(3):310-6.

45. Ibrahim LF, Hopper SM, Orsini F, Daley AJ, Babl FE, Bryant PA. Efficacy and safety of intravenous ceftriaxone at home versus intravenous flucloxacillin in hospital for children with cellulitis (CHOICE): a single-centre, open-label, randomised, controlled, non-inferiority trial. The Lancet Infectious Diseases. 2019 May 1;19(5):477-86.

46. Solomkin J, Zhao YP, Ma EL, Chen MJ, Hampel B, DRAGON Study Team. Moxifloxacin is non-inferior to combination therapy with ceftriaxone plus metronidazole in patients with community-origin complicated intra-abdominal infections. International journal of antimicrobial agents. 2009 Nov 1;34(5):439-45.

47. Lv X, Alder J, Li L, O’Riordan W, Rybak MJ, Ye H, Zhang R, Zhang Z, Zhu X, Wilcox MH. Efficacy and safety of tedizolid phosphate versus linezolid in a randomized phase 3 trial in patients with acute bacterial skin and skin structure infection. Antimicrobial agents and chemotherapy. 2019 Jul 1;63(7):e02252-18.

48. Yuan J, Mo B, Ma Z, Lv Y, Cheng SL, Yang Y, Tong Z, Wu R, Sun S, Cao Z, Wu J. Safety and efficacy of oral nemonoxacin versus levofloxacin in treatment of community-acquired pneumonia: A phase 3, multicenter, randomized, double-blind, double-dummy, active-controlled, non-inferiority trial. Journal of Microbiology, Immunology and Infection. 2019 Feb 1;52(1):35-44.

49. Liou JM, Chen CC, Fang YJ, Chen PY, Chang CY, Chou CK, Chen MJ, Tseng CH, Lee JY, Yang TH, Chiu MC. 14 day sequential therapy versus 10 day bismuth quadruple therapy containing high-dose esomeprazole in the first-line and second-line treatment of Helicobacter pylori: a multicentre, non-inferiority, randomized trial. Journal of Antimicrobial Chemotherapy. 2018 Sep 1;73(9):2510-8.

50. Aseffa A, Chukwu JN, Vahedi M, Aguwa EN, Bedru A, Mebrahtu T, Ezechi OC, Yimer G, Yamuah LK, Medhin G, Connolly C. Efficacy and safety of ‘fixed dose’versus ‘loose’drug regimens for treatment of pulmonary tuberculosis in two high TB-burden African countries: a randomized controlled trial. PLoS One. 2016;11(6).

51. File Jr TM, Schlemmer B, Garau J, Cupo M, Young C, 049 Clinical Study Group. Efficacy and safety of gemifloxacin in the treatment of community-acquired pneumonia: a randomized, double-blind comparison with trovafloxacin. Journal of Antimicrobial Chemotherapy. 2001 Jul 1;48(1):67-74.

52. Harris PN, Tambyah PA, Lye DC, Mo Y, Lee TH, Yilmaz M, Alenazi TH, Arabi Y, Falcone M, Bassetti M, Righi E. Effect of piperacillin-tazobactam vs meropenem on 30-day mortality for patients with E coli or Klebsiella pneumoniae bloodstream infection and ceftriaxone resistance: a randomized clinical trial. Jama. 2018 Sep 11;320(10):984-94.

53. Aliberti S, Ramirez J, Giuliani F, Wiemken T, Sotgiu G, Tedeschi S, Carugati M, Valenti V, Marchioni M, Camera M, Piro R. Individualizing duration of antibiotic therapy in community-acquired pneumonia. Pulmonary pharmacology & therapeutics. 2017 Aug 1;45:191-201.

54. Giusti M, Blasi F, Iori I, Mazzone A, Sgambato F, Politi C, Colagrande P, Casali A, Valerio A, Gussoni G, Bonizzoni E. Prulifloxacin vs Levofloxacin for exacerbation of COPD after failure of other antibiotics. COPD: Journal of Chronic Obstructive Pulmonary Disease. 2016 Sep 2;13(5):555-60.

55. Oliva ME, Rekha A, Yellin A, Pasternak J, Campos M, Rose GM, Babinchak T, Ellis-Grosse EJ, Loh E, 301 Study Group. A multicenter trial of the efficacy and safety of tigecycline versus imipenem/cilastatin in patients with complicated intra-abdominal infections [Study ID Numbers: 3074A1-301-WW; ClinicalTrials. gov Identifier: NCT00081744]. BMC infectious diseases. 2005 Dec 1;5(1):88.

56. Judlin P, Liao Q, Liu Z, Reimnitz P, Hampel B, Arvis P. Efficacy and safety of moxifloxacin in uncomplicated pelvic inflammatory disease: the MONALISA study. BJOG: An International Journal of Obstetrics & Gynaecology. 2010 Nov;117(12):1475-84.

57. Zhang W, Chen Q, Liang X, Liu W, Xiao S, Graham DY, Lu H. Bismuth, lansoprazole, amoxicillin and metronidazole or clarithromycin as first-line Helicobacter pylori therapy. Gut. 2015 Nov 1;64(11):1715-20.

58. Ross JD, Cronje HS, Paszkowski T, Rakoczi I, Vildaite D, Kureishi A, Alefelder M, Arvis P, Reimnitz P. Moxifloxacin versus ofloxacin plus metronidazole in uncomplicated pelvic inflammatory disease: results of a multicentre, double blind, randomised trial. Sexually transmitted infections. 2006 Dec 1;82(6):446-51.

59. Nicholson SC, Welte T, File Jr TM, Strauss RS, Michiels B, Kaul P, Balis D, Arbit D, Amsler K, Noel GJ. A randomised, double-blind trial comparing ceftobiprole medocaril with ceftriaxone with or without linezolid for the treatment of patients with community-acquired pneumonia requiring hospitalisation. International journal of antimicrobial agents. 2012 Mar 1;39(3):240-6.

60. Wilson R, Anzueto A, Miravitlles M, Arvis P, Alder J, Haverstock D, Trajanovic M, Sethi S. Moxifloxacin versus amoxicillin/clavulanic acid in outpatient acute exacerbations of COPD: MAESTRAL results. European Respiratory Journal. 2012 Jul 1;40(1):17-27.

61. Rob F, Klubalová B, Nyčová E, Hercogová J, Unemo M. Gentamicin 240 mg plus azithromycin 2 g vs. ceftriaxone 500 mg plus azithromycin 2 g for treatment of rectal and pharyngeal gonorrhoea: a randomized controlled trial. Clinical Microbiology and Infection. 2020 Feb 1;26(2):207-12.

62. Petitpretz P, Choné C, Trémolières F, Investigator Study Group. Levofloxacin 500 mg once daily versus cefuroxime 250 mg twice daily in patients with acute exacerbations of chronic obstructive bronchitis: clinical efficacy and exacerbation-free interval. International journal of antimicrobial agents. 2007 Jul 1;30(1):52-9.

63. el Moussaoui R, de Borgie CA, van den Broek P, Hustinx WN, Bresser P, van den Berk GE, Poley JW, van den Berg B, Krouwels FH, Bonten MJ, Weenink C. Effectiveness of discontinuing antibiotic treatment after three days versus eight days in mild to moderate-severe community acquired pneumonia: randomised, double blind study.

64. Fabian TC, File Jr TM, Embil JM, Krige JE, Klein S, Rose A, Melnick D, Soto NE. Meropenem versus imipenem-cilastatin for the treatment of hospitalized patients with complicated skin and skin structure infections: results of a multicenter, randomized, double-blind comparative study. Surgical infections. 2005 Sep 1;6(3):269-82.

65. Siquier, B., Sanchez-Alvarez, J., Garcia-Mendez, E., Sabriá, M., Santos, J., Pallarés, R., Twynholm, M. and Dal-Re, R., 2006. Efficacy and safety of twice-daily pharmacokinetically enhanced amoxicillin/clavulanate (2000/125 mg) in the treatment of adults with community-acquired pneumonia in a country with a high prevalence of penicillin-resistant Streptococcus pneumoniae. *Journal of Antimicrobial Chemotherapy*, *57*(3), pp.536-545.

66. Corey GR, Wilcox MH, Talbot GH, Thye D, Friedland D, Baculik T, CANVAS 1 investigators, Mehra P, Alpert M, Baird I, Klein S. CANVAS 1: the first Phase III, randomized, double-blind study evaluating ceftaroline fosamil for the treatment of patients with complicated skin and skin structure infections. Journal of antimicrobial chemotherapy. 2010 Nov 1;65(suppl_4):iv41-51.

67. Wilcox MH, Corey GR, Talbot GH, Thye D, Friedland D, Baculik T, CANVAS 2 investigators, Manos P, Lee P, Bush L, DeSanto J. CANVAS 2: the second Phase III, randomized, double-blind study evaluating ceftaroline fosamil for the treatment of patients with complicated skin and skin structure infections. Journal of antimicrobial chemotherapy. 2010 Nov 1;65(suppl_4):iv53-65.

68. File Jr TM, Mandell LA, Tillotson G, Kostov K, Georgiev O. Gemifloxacin once daily for 5 days versus 7 days for the treatment of community-acquired pneumonia: a randomized, multicentre, double-blind study. Journal of Antimicrobial Chemotherapy. 2007 Jul 1;60(1):112-20.

69. File Jr TM, Low DE, Eckburg PB, Talbot GH, Friedland HD, Lee J, Llorens L, Critchley IA, Thye DA, Focus 1 Investigators, Pullman J. FOCUS 1: a randomized, double-blinded, multicentre, phase III trial of the efficacy and safety of ceftaroline fosamil versus ceftriaxone in community-acquired pneumonia. Journal of antimicrobial chemotherapy. 2011 Apr 1;66(suppl_3):iii19-32.

70. Low DE, File Jr TM, Eckburg PB, Talbot GH, David Friedland H, Lee J, Llorens L, Critchley IA, Thye DA, Focus 2 Investigators, Corral J. FOCUS 2: a randomized, double-blinded, multicentre, Phase III trial of the efficacy and safety of ceftaroline fosamil versus ceftriaxone in community-acquired pneumonia. Journal of antimicrobial chemotherapy. 2011 Apr 1;66(suppl_3):iii33-44.

71. Huang, D.B., O’Riordan, W., Overcash, J.S., Heller, B., Amin, F., File, T.M., Wilcox, M.H., Torres, A., Dryden, M., Holland, T.L. and McLeroth, P., 2018. A phase 3, randomized, double-blind, multicenter study to evaluate the safety and efficacy of intravenous iclaprim vs vancomycin for the treatment of acute bacterial skin and skin structure infections suspected or confirmed to be due to gram-positive pathogens: REVIVE-1. *Clinical Infectious Diseases*, *66*(8), pp.1222-1229.

72. Corey GR, Kabler H, Mehra P, Gupta S, Overcash JS, Porwal A, Giordano P, Lucasti C, Perez A, Good S, Jiang H. Single-dose oritavancin in the treatment of acute bacterial skin infections. New England Journal of Medicine. 2014 Jun 5;370(23):2180-90.

73. Stets R, Popescu M, Gonong JR, Mitha I, Nseir W, Madej A, Kirsch C, Das AF, Garrity-Ryan L, Steenbergen JN, Manley A. Omadacycline for community-acquired bacterial pneumonia. New England Journal of Medicine. 2019 Feb 7;380(6):517-27.

74. O'Riordan W, Cardenas C, Shin E, Sirbu A, Garrity-Ryan L, Das AF, Eckburg PB, Manley A, Steenbergen JN, Tzanis E, McGovern PC. Once-daily oral omadacycline versus twice-daily oral linezolid for acute bacterial skin and skin structure infections (OASIS-2): a phase 3, double-blind, multicentre, randomised, controlled, non-inferiority trial. The Lancet Infectious Diseases. 2019 Oct 1;19(10):1080-90.

75. Federico A, Nardone G, Gravina AG, Iovene MR, Miranda A, Compare D, Pilloni PA, Rocco A, Ricciardiello L, Marmo R, Loguercio C. Efficacy of 5-day levofloxacin-containing concomitant therapy in eradication of Helicobacter pylori infection. Gastroenterology. 2012 Jul 1;143(1):55-61.

76. Freire AT, Melnyk V, Kim MJ, Datsenko O, Dzyublik O, Glumcher F, Chuang YC, Maroko RT, Dukart G, Cooper CA, Korth-Bradley JM. Comparison of tigecycline with imipenem/cilastatin for the treatment of hospital-acquired pneumonia. Diagnostic microbiology and infectious disease. 2010 Oct 1;68(2):140-51.

77. Lauf L, Ozsvár Z, Mitha I, Regöly-Mérei J, Embil JM, Cooper A, Sabol MB, Castaing N, Dartois N, Yan J, Dukart G. Phase 3 study comparing tigecycline and ertapenem in patients with diabetic foot infections with and without osteomyelitis. Diagnostic microbiology and infectious disease. 2014 Apr 1;78(4):469-80.

78. Dartois N, Castaing N, Gandjini H, Cooper A. Tigecycline versus levofloxacin for the treatment of community-acquired pneumonia: European experience. Journal of Chemotherapy. 2008 Sep 1;20(sup1):28-35.

79. Bergallo C, Jasovich A, Teglia O, Oliva ME, Lentnek A, de Wouters L, Zlocowski JC, Dukart G, Cooper A, Mallick R, 308 Study Group. Safety and efficacy of intravenous tigecycline in treatment of community-acquired pneumonia: results from a double-blind randomized phase 3 comparison study with levofloxacin. Diagnostic microbiology and infectious disease. 2009 Jan 1;63(1):52-61.

80. Sacchidanand S, Penn RL, Embil JM, Campos ME, Curcio D, Ellis-Grosse E, Loh E, Rose G. Efficacy and safety of tigecycline monotherapy compared with vancomycin plus aztreonam in patients with complicated skin and skin structure infections: results from a phase 3, randomized, double-blind trial. International journal of infectious diseases. 2005 Sep 1;9(5):251-61.

81. Louie TJ, Miller MA, Mullane KM, Weiss K, Lentnek A, Golan Y, Gorbach S, Sears P, Shue YK. Fidaxomicin versus vancomycin for Clostridium difficile infection. New England Journal of Medicine. 2011 Feb 3;364(5):422-31.

82. Solomkin J, Hershberger E, Miller B, Popejoy M, Friedland I, Steenbergen J, Yoon M, Collins S, Yuan G, Barie PS, Eckmann C. Ceftolozane/tazobactam plus metronidazole for complicated intra-abdominal infections in an era of multidrug resistance: results from a randomized, double-blind, phase 3 trial (ASPECT-cIAI). Clinical Infectious Diseases. 2015 May 15;60(10):1462-71.

83. Weiss G, Reimnitz P, Hampel B, Muehlhofer E, Lippert H. Moxifloxacin for the treatment of patients with complicated intra-abdominal infections (the AIDA Study). Journal of Chemotherapy. 2009 Apr 1;21(2):170-80.

84. Molina–Infante J, Romano M, Fernandez–Bermejo M, Federico A, Gravina AG, Pozzati L, Garcia–Abadia E, Vinagre–Rodriguez G, Martinez–Alcala C, Hernandez–Alonso M, Miranda A. Optimized nonbismuth quadruple therapies cure most patients with Helicobacter pylori infection in populations with high rates of antibiotic resistance. Gastroenterology. 2013 Jul 1;145(1):121-8.

85. Mazuski JE, Gasink LB, Armstrong J, Broadhurst H, Stone GG, Rank D, Llorens L, Newell P, Pachl J. Efficacy and safety of ceftazidime-avibactam plus metronidazole versus meropenem in the treatment of complicated intra-abdominal infection: results from a randomized, controlled, double-blind, phase 3 program. Clinical Infectious Diseases. 2016 Jun 1;62(11):1380-9.

86. Chung JW, Lee JH, Jung HY, Yun SC, Oh TH, Choi KD, Song HJ, Lee GH, Kim JH. Second‐line Helicobacter pylori eradication: a randomized comparison of 1‐week or 2‐week bismuth‐containing quadruple therapy. Helicobacter. 2011 Aug;16(4):289-94.

87. Dore MP, Farina V, Cuccu M, Mameli L, Massarelli G, Graham DY. Twice‐a‐day bismuth‐containing quadruple therapy for Helicobacter pylori eradication: a randomized trial of 10 and 14 days. Helicobacter. 2011 Aug;16(4):295-300.

88. Tsay FW, Wu DC, Kao SS, Tsai TJ, Lai KH, Cheng JS, Chan HH, Wang HM, Tsai WL, Tseng HH, Peng NJ. Reverse Sequential Therapy Achieves a Similar Eradication Rate as Standard Sequential Therapy for H elicobacter pylori Eradication: A Randomized Controlled Trial. Helicobacter. 2015 Feb;20(1):71-7.

89. Bohbot JM, Vicaut E, Fagnen D, Brauman M. Treatment of bacterial vaginosis: a multicenter, double-blind, double-dummy, randomised phase III study comparing secnidazole and metronidazole. Infectious diseases in obstetrics and gynecology. 2010;2010.

90. Jindani A, Harrison TS, Nunn AJ, Phillips PP, Churchyard GJ, Charalambous S, Hatherill M, Geldenhuys H, McIlleron HM, Zvada SP, Mungofa S. High-dose rifapentine with moxifloxacin for pulmonary tuberculosis. N Engl J Med. 2014 Oct 23;371:1599-608.

91. Lienhardt C, Cook SV, Burgos M, Yorke-Edwards V, Rigouts L, Anyo G, Kim SJ, Jindani A, Enarson DA, Nunn AJ, Study C Trial Group. Efficacy and safety of a 4-drug fixed-dose combination regimen compared with separate drugs for treatment of pulmonary tuberculosis: the Study C randomized controlled trial. Jama. 2011 Apr 13;305(14):1415-23.

92. Merle CS, Fielding K, Sow OB, Gninafon M, Lo MB, Mthiyane T, Odhiambo J, Amukoye E, Bah B, Kassa F, N'Diaye A. A four-month gatifloxacin-containing regimen for treating tuberculosis. New England Journal of Medicine. 2014 Oct 23;371(17):1588-98.

93. Gillespie SH, Crook AM, McHugh TD, Mendel CM, Meredith SK, Murray SR, Pappas F, Phillips PP, Nunn AJ. Four-month moxifloxacin-based regimens for drug-sensitive tuberculosis. New England Journal of Medicine. 2014 Oct 23;371(17):1577-87.

94. Nunn AJ, Phillips PP, Meredith SK, Chiang CY, Conradie F, Dalai D, Van Deun A, Dat PT, Lan N, Master I, Mebrahtu T. A trial of a shorter regimen for rifampin-resistant tuberculosis. New England Journal of Medicine. 2019 Mar 28;380(13):1201-13.

95. Kaye KS, Bhowmick T, Metallidis S, Bleasdale SC, Sagan OS, Stus V, Vazquez J, Zaitsev V, Bidair M, Chorvat E, Dragoescu PO. Effect of meropenem-vaborbactam vs piperacillin-tazobactam on clinical cure or improvement and microbial eradication in complicated urinary tract infection: the TANGO I randomized clinical trial. Jama. 2018 Feb 27;319(8):788-99.

96. File TM, Lode H, Kurz H, Kozak R, Xie H, Berkowitz E. Double-blind, randomized study of the efficacy and safety of oral pharmacokinetically enhanced amoxicillin-clavulanate (2,000/125 milligrams) versus those of amoxicillin-clavulanate (875/125 milligrams), both given twice daily for 7 days, in treatment of bacterial community-acquired pneumonia in adults. Antimicrobial agents and chemotherapy. 2004 Sep 1;48(9):3323-31.

97. van Rensburg DJ, Perng RP, Mitha IH, Bester AJ, Kasumba J, Wu RG, Ho ML, Chang LW, Chung DT, Chang YT, King CH. Efficacy and safety of nemonoxacin versus levofloxacin for community-acquired pneumonia. Antimicrobial agents and chemotherapy. 2010 Oct 1;54(10):4098-106.

98. Desrosiers M, Ferguson B, Michel Klossek J, Drugeon H, Mösges R. Clinical efficacy and time to symptom resolution of 5-day telithromycin versus 10-day amoxicillin–clavulanate in the treatment of acute bacterial sinusitis. Current medical research and opinion. 2008 Jun 1;24(6):1691-702.

99. Lucasti C, Jasovich A, Umeh O, Jiang J, Kaniga K, Friedland I. Efficacy and tolerability of IV doripenem versus meropenem in adults with complicated intra-abdominal infection: a phase III, prospective, multicenter, randomized, double-blind, noninferiority study. Clinical therapeutics. 2008 May 1;30(5):868-83.

100. Corey GR, Good S, Jiang H, Moeck G, Wikler M, Green S, Manos P, Keech R, Singh R, Heller B, Bubnova N. Single-dose oritavancin versus 7–10 days of vancomycin in the treatment of gram-positive acute bacterial skin and skin structure infections: the SOLO II noninferiority study. Clinical Infectious Diseases. 2015 Jan 15;60(2):254-62.

101. Clegg HW, Ryan AG, Dallas SD, Kaplan EL, Johnson DR, Norton HJ, Roddey OF, Martin ES, Swetenburg RL, Koonce EW, Felkner MM. Treatment of streptococcal pharyngitis with once-daily compared with twice-daily amoxicillin: a noninferiority trial. The Pediatric infectious disease journal. 2006 Sep 1;25(9):761-7.

102. Zanetti G, Bally F, Greub G, Garbino J, Kinge T, Lew D, Romand JA, Bille J, Aymon D, Stratchounski L, Krawczyk L. Cefepime versus imipenem-cilastatin for treatment of nosocomial pneumonia in intensive care unit patients: a multicenter, evaluator-blind, prospective, randomized study. Antimicrobial agents and chemotherapy. 2003 Nov 1;47(11):3442-7.

103. Rimoin AW, Hoff NA, Fischer Walker CL, Hamza HS, Vince A, Rahman NA, Andrasevic S, Emam S, Vukelic D, Elminawi N, Ghafar HA. Treatment of streptococcal pharyngitis with once-daily amoxicillin versus intramuscular benzathine penicillin G in low-resource settings: a randomized controlled trial. Clinical pediatrics. 2011 Jun;50(6):535-42.

104. Uranga A, España PP, Bilbao A, Quintana JM, Arriaga I, Intxausti M, Lobo JL, Tomás L, Camino J, Nuñez J, Capelastegui A. Duration of antibiotic treatment in community-acquired pneumonia: a multicenter randomized clinical trial. JAMA internal medicine. 2016 Sep 1;176(9):1257-65.

105. Li HK, Rombach I, Zambellas R, Walker AS, McNally MA, Atkins BL, Lipsky BA, Hughes HC, Bose D, Kümin M, Scarborough C. Oral versus intravenous antibiotics for bone and joint infection. New England Journal of Medicine. 2019 Jan 31;380(5):425-36.

106. Naber KG, Eisenstein BI, Tally FP. Daptomycin versus ciprofloxacin in the treatment of complicated urinary tract infection due to Gram-positive bacteria. Infectious Diseases in Clinical Practice. 2004 Nov 1;12(6):322-7.

107. Noel GJ, Strauss RS, Amsler K, Heep M, Pypstra R, Solomkin JS. Results of a double-blind, randomized trial of ceftobiprole treatment of complicated skin and skin structure infections caused by gram-positive bacteria. Antimicrobial agents and chemotherapy. 2008 Jan 1;52(1):37-44.

108. UpChurch J, Rosemore M, Tosiello R, Kowalsky S, Echols R. Randomized double-blind study comparing 7-and 10-day regimens of faropenem medoxomil with a 10-day cefuroxime axetil regimen for treatment of acute bacterial sinusitis. Otolaryngology--Head and Neck Surgery. 2006 Oct;135(4):511-7.

109. O’Riordan W, McManus A, Teras J, Poromanski I, Cruz-Saldariagga M, Quintas M, Lawrence L, Liang S, Cammarata S. A comparison of the efficacy and safety of intravenous followed by oral delafloxacin with vancomycin plus aztreonam for the treatment of acute bacterial skin and skin structure infections: a phase 3, multinational, double-blind, randomized study. Clinical Infectious Diseases. 2018 Aug 16;67(5):657-66.

110. Chastre J, Wunderink R, Prokocimer P, Lee M, Kaniga K, Friedland I. Efficacy and safety of intravenous infusion of doripenem versus imipenem in ventilator-associated pneumonia: a multicenter, randomized study. Critical care medicine. 2008 Apr 1;36(4):1089-96.

111. Leroy O, Saux P, Bédos JP, Caulin E, Levofloxacin Study Group. Comparison of levofloxacin and cefotaxime combined with ofloxacin for ICU patients with community-acquired pneumonia who do not require vasopressors. Chest. 2005 Jul 1;128(1):172-83.

112. Torres A, Garau J, Arvis P, Carlet J, Choudhri S, Kureishi A, Le Berre MA, Lode H, Winter J, Read RC, MOTIV (MOxifloxacin Treatment IV) Study Group. Moxifloxacin monotherapy is effective in hospitalized patients with community-acquired pneumonia: the MOTIV study—a randomized clinical trial. Clinical infectious diseases. 2008 May 15;46(10):1499-509.

113. Agweyu A, Gathara D, Oliwa J, Muinga N, Edwards T, Allen E, Maleche-Obimbo E, English M, Severe Pneumonia Study Group, Aweyo F, Awuonda B. Oral amoxicillin versus benzyl penicillin for severe pneumonia among kenyan children: a pragmatic randomized controlled noninferiority trial. Clinical Infectious Diseases. 2015 Apr 15;60(8):1216-24.

114. Lojanapiwat B, Nimitvilai S, Bamroongya M, Jirajariyavej S, Tiradechavat C, Malithong A, Predanon C, Tanphaichitra D, Lertsupphakul B. Oral sitafloxacin vs intravenous ceftriaxone followed by oral cefdinir for acute pyelonephritis and complicated urinary tract infection: a randomized controlled trial. Infection and drug resistance. 2019;12:173.

115. Iversen K, Ihlemann N, Gill SU, Madsen T, Elming H, Jensen KT, Bruun NE, Høfsten DE, Fursted K, Christensen JJ, Schultz M. Partial oral versus intravenous antibiotic treatment of endocarditis. New England Journal of Medicine. 2019 Jan 31;380(5):415-24.

116. Solomkin J, Evans D, Slepavicius A, Lee P, Marsh A, Tsai L, Sutcliffe JA, Horn P. Assessing the efficacy and safety of eravacycline vs ertapenem in complicated intra-abdominal infections in the Investigating Gram-Negative Infections Treated With Eravacycline (IGNITE 1) trial: a randomized clinical trial. JAMA surgery. 2017 Mar 1;152(3):224-32.

117. Awad SS, Rodriguez AH, Chuang YC, Marjanek Z, Pareigis AJ, Reis G, Scheeren TW, Sánchez AS, Zhou X, Saulay M, Engelhardt M. A phase 3 randomized double-blind comparison of ceftobiprole medocaril versus ceftazidime plus linezolid for the treatment of hospital-acquired pneumonia. Clinical infectious diseases. 2014 Jul 1;59(1):51-61.

118. Bradley JS, Arguedas A, Blumer JL, Sáez-Llorens X, Melkote R, Noel GJ. Comparative study of levofloxacin in the treatment of children with community-acquired pneumonia. The Pediatric infectious disease journal. 2007 Oct 1;26(10):868-78.

119. Klausner HA, Brown P, Peterson J, Kaul S, Khashab M, Fisher AC, Kahn JB. A trial of levofloxacin 750 mg once daily for 5 days versus ciprofloxacin 400 mg and/or 500 mg twice daily for 10 days in the treatment of acute pyelonephritis. Current medical research and opinion. 2007 Nov 1;23(11):2637-45.

120. Fowler Jr VG, Boucher HW, Corey GR, Abrutyn E, Karchmer AW, Rupp ME, Levine DP, Chambers HF, Tally FP, Vigliani GA, Cabell CH. Daptomycin versus standard therapy for bacteremia and endocarditis caused by Staphylococcus aureus. New England Journal of Medicine. 2006 Aug 17;355(7):653-65.

121. Saar S, Mihnovitš V, Lustenberger T, Rauk M, Noor EH, Lipping E, Isand KG, Lepp J, Lomp A, Lepner U, Talving P. Twenty-four hour versus extended antibiotic administration after surgery in complicated appendicitis: A randomized controlled trial. Journal of Trauma and Acute Care Surgery. 2019 Jan 1;86(1):36-42.

122. Fourcroy JL, Berner B, Chiang YK, Cramer M, Rowe L, Shore N. Efficacy and safety of a novel once-daily extended-release ciprofloxacin tablet formulation for treatment of uncomplicated urinary tract infection in women. Antimicrobial agents and chemotherapy. 2005 Oct 1;49(10):4137-43.

123. Hooton TM, Roberts PL, Stapleton AE. Cefpodoxime vs ciprofloxacin for short-course treatment of acute uncomplicated cystitis: a randomized trial. Jama. 2012 Feb 8;307(6):583-9.

124. Noel GJ, Bush K, Bagchi P, Ianus J, Strauss RS. A randomized, double-blind trial comparing ceftobiprole medocaril with vancomycin plus ceftazidime for the treatment of patients with complicated skin and skin-structure infections. Clinical infectious diseases. 2008 Mar 1;46(5):647-55.

125. Peterson J, Kaul S, Khashab M, Fisher AC, Kahn JB. A double-blind, randomized comparison of levofloxacin 750 mg once-daily for five days with ciprofloxacin 400/500 mg twice-daily for 10 days for the treatment of complicated urinary tract infections and acute pyelonephritis. Urology. 2008 Jan 1;71(1):17-22.

126. English ML, Fredericks CE, Milanesio NA, Rohowsky N, Xu ZQ, Jenta TR, Flavin MT, Eiznhamer DA. Cethromycin versus clarithromycin for community-acquired pneumonia: comparative efficacy and safety outcomes from two double-blinded, randomized, parallel-group, multicenter, multinational noninferiority studies. Antimicrobial agents and chemotherapy. 2012 Apr 1;56(4):2037-47.

127. Rubinstein E, Lalani T, Corey GR, Kanafani ZA, Nannini EC, Rocha MG, Rahav G, Niederman MS, Kollef MH, Shorr AF, Lee PC. Telavancin versus vancomycin for hospital-acquired pneumonia due to gram-positive pathogens. Clinical Infectious Diseases. 2011 Jan 1;52(1):31-40.

128. Mir MA, Chaudhary S, Payasi A, Sood R, Mavuduru RS, Shameem M. Ceftriaxone+ Sulbactam+ Disodium EDTA Versus Meropenem for the Treatment of Complicated Urinary Tract Infections, Including Acute Pyelonephritis: PLEA, a Double-Blind, Randomized Noninferiority Trial. InOpen forum infectious diseases 2019 Oct (Vol. 6, No. 10, p. ofz373). US: Oxford University Press.

129. Solomkin JS, Gardovskis J, Lawrence K, Montravers P, Sway A, Evans D, Tsai L. IGNITE4: results of a phase 3, randomized, multicenter, prospective trial of eravacycline vs meropenem in the treatment of complicated intraabdominal infections. Clinical Infectious Diseases. 2019 Aug 30;69(6):921-9.

130. Chen Y, Zhu D, Zhang Y, Zhao Y, Chen G, Li P, Xu L, Yan P, Hickman MA, Xu X, Tawadrous M. A multicenter, double-blind, randomized, comparison study of the efficacy and safety of tigecycline to imipenem/cilastatin to treat complicated intra-abdominal infections in hospitalized subjects in China. Therapeutics and clinical risk management. 2018;14:2327.

131. Sapmaz F, Kalkan IH, Atasoy P, Basyigit S, Guliter S. A Non-Inferiority Study: Modified Dual Therapy Consisting Higher Doses of Rabeprazole Is as Successful as Standard Quadruple Therapy in Eradication of: Helicobacter pylori. American journal of therapeutics. 2017 Jul 1;24(4):e393-8.

132. Cao C, Luo A, Wu P, Weng D, Zheng H, Wang S. Efficacy and safety of morinidazole in pelvic inflammatory disease: results of a multicenter, double-blind, randomized trial. European Journal of Clinical Microbiology & Infectious Diseases. 2017 Jul 1;36(7):1225-30.

133. Köksal AS, Onder FO, Torun S, Parlak E, Sayilir A, Tayfur O, Yildiz H, Ozbalci GS, Kayaçetin E. Twice a day quadruple therapy for the first-line treatment of Helicobacter pylori in an area with a high prevalence of background antibiotic resistance. Acta gastro-enterologica Belgica. 2013 Jan;76(1):34-7.

134. Molton JS, Chan M, Kalimuddin S, Oon J, Young BE, Low JG, Salada B, Lee TH, Wijaya L, Fisher DA, Izharuddin E. Oral vs Intravenous Antibiotics for Patients With Klebsiella pneumoniae Liver Abscess: A Randomized, Controlled Noninferiority Study. Clinical Infectious Diseases. 2019 Oct 23.

135. Cranendonk DR, Opmeer BC, van Agtmael MA, Branger J, Brinkman K, Hoepelman AI, Lauw FN, Oosterheert JJ, Pijlman AH, Sankatsing SU, Soetekouw R. Antibiotic treatment for 6 days versus 12 days in patients with severe cellulitis: a multicentre randomized, double-blind, placebo-controlled, non-inferiority trial. Clinical Microbiology and Infection. 2019 Oct 13.

136. File Jr TM, Goldberg L, Das A, Sweeney C, Saviski J, Gelone SP, Seltzer E, Paukner S, Wicha WW, Talbot GH, Gasink LB. Efficacy and safety of intravenous-to-oral lefamulin, a pleuromutilin antibiotic, for the treatment of community-acquired bacterial pneumonia: the phase III Lefamulin Evaluation Against Pneumonia (LEAP 1) trial. Clinical Infectious Diseases. 2019 Nov 13;69(11):1856-67.

137. Clarke MC, Cheng AC, Pollard JG, Birch M, Cowan RU, Linke JA, Walton AL, Friedman ND. Lessons Learned From a Randomized Controlled Trial of Short-Course Intravenous Antibiotic Therapy for Erysipelas and Cellulitis of the Lower Limb (Switch Trial). InOpen forum infectious diseases 2019 Sep (Vol. 6, No. 9, p. ofz335). US: Oxford University Press.

138. Yahav D, Franceschini E, Koppel F, Turjeman A, Babich T, Bitterman R, Neuberger A, Ghanem-Zoubi N, Santoro A, Eliakim-Raz N, Pertzov B. Seven versus 14 days of antibiotic therapy for uncomplicated gram-negative bacteremia: a noninferiority randomized controlled trial. Clinical Infectious Diseases. 2019 Sep 13;69(7):1091-8.

139. Munteanu D, Etzion O, Ben-Yakov G, Halperin D, Eidelman L, Schwartz D, Novack V, Abufreha N, Krugliak P, Rozenthal A, Gaspar N. Efficacy and safety of sequential versus quadruple therapy as second-line treatment for helicobacter pylori infection—A randomized controlled trial. PloS one. 2017;12(9).

140. Tancawan AL, Pato MN, Abidin KZ, Asari AS, Thong TX, Kochhar P, Muganurmath C, Twynholm M, Barker K. Amoxicillin/clavulanic acid for the treatment of odontogenic infections: a randomised study comparing efficacy and tolerability versus clindamycin. International journal of dentistry. 2015;2015.

141. Fomin P, Beuran M, Gradauskas A, Barauskas G, Datsenko A, Dartois N, Ellis-Grosse E, Loh E. Tigecycline is efficacious in the treatment of complicated intra-abdominal infections. International Journal of Surgery. 2005 Jan 1;3(1):35-47.

142. O’Riordan W, Green S, Overcash JS, Puljiz I, Metallidis S, Gardovskis J, Garrity-Ryan L, Das AF, Tzanis E, Eckburg PB, Manley A. Omadacycline for acute bacterial skin and skin-structure infections. New England Journal of Medicine. 2019 Feb 7;380(6):528-38.

143. Graham DR, Christopher L, Osvaldo M, Nichols RL, Paul H, Perez NQ, McAdams A, Woods GL, Ceesay TP, Gesser R, Ertapenem Complicated Skin and Skin Structure Infections Study Group. Ertapenem once daily versus piperacillin-tazobactam 4 times per day for treatment of complicated skin and skin-structure infections in adults: results of a prospective, randomized, double-blind multicenter study. Clinical infectious diseases. 2002 Jun 1;34(11):1460-8.

144. Lipsky BA, Armstrong DG, Citron DM, Tice AD, Morgenstern DE, Abramson MA. Ertapenem versus piperacillin/tazobactam for diabetic foot infections (SIDESTEP): prospective, randomised, controlled, double-blinded, multicentre trial. The Lancet. 2005 Nov 12;366(9498):1695-703.

145. Ortiz-Ruiz G, Caballero-Lopez J, Friedland IR, Woods GL, Carides A, Protocol 018 Ertapenem Community-Acquired Pneumonia Study Group. A study evaluating the efficacy, safety, and tolerability of ertapenem versus ceftriaxone for the treatment of community-acquired pneumonia in adults. Clinical infectious diseases. 2002 Apr 15;34(8):1076-83.

146. Vetter N, Cambronero-Hernandez E, Rohlf J, Simon S, Carides A, Oliveria T, Isaacs R, Protocol 020 Study Group. A prospective, randomized, double-blind multicenter comparison of parenteral ertapenem and ceftriaxone for the treatment of hospitalized adults with community-acquired pneumonia. Clinical therapeutics. 2002 Nov 1;24(11):1770-85.

147. Jimenez-Cruz F, Jasovich A, Cajigas J, Jiang Q, Imbeault D, Woods GL, Gesser RM, Protocol 021 Study Group. A prospective, multicenter, randomized, double-blind study comparing ertapenem and ceftriaxone followed by appropriate oral therapy for complicated urinary tract infections in adults. Urology. 2002 Jul 1;60(1):16-22.

148. Tomera KM, Burdmann EA, Reyna OG, Jiang Q, Wimmer WM, Woods GL, Gesser RM. Ertapenem versus ceftriaxone followed by appropriate oral therapy for treatment of complicated urinary tract infections in adults: results of a prospective, randomized, double-blind multicenter study. Antimicrobial agents and chemotherapy. 2002 Sep 1;46(9):2895-900.

149. Roy S, Higareda I, Angel-Muller E, Ismail M, Hague C, Adeyi B, Woods GL, Teppler H. Ertapenem once a day versus piperacillin–tazobactam every 6 hours for treatment of acute pelvic infections: a prospective, multicenter, randomized, double-blind study. Infectious diseases in obstetrics and gynecology. 2003;11(1):27-37.

150. Tellier G, Niederman MS, Nusrat R, Patel M, Lavin B. Clinical and bacteriological efficacy and safety of 5 and 7 day regimens of telithromycin once daily compared with a 10 day regimen of clarithromycin twice daily in patients with mild to moderate community-acquired pneumonia. Journal of Antimicrobial Chemotherapy. 2004 Aug 1;54(2):515-23.

151. Dunbar LM, Hassman J, Tellier G. Efficacy and tolerability of once-daily oral telithromycin compared with clarithromycin for the treatment of community-acquired pneumonia in adults. Clinical therapeutics. 2004 Jan 1;26(1):48-62.

152. Stryjewski ME, Graham DR, Wilson SE, O'Riordan W, Young D, Lentnek A, Ross DP, Fowler VG, Hopkins A, Friedland HD, Barriere SL. Telavancin versus vancomycin for the treatment of complicated skin and skin-structure infections caused by gram-positive organisms. Clinical infectious diseases. 2008 Jun 1;46(11):1683-93.

153. Naber KG, Llorens L, Kaniga K, Kotey P, Hedrich D, Redman R. Intravenous doripenem at 500 milligrams versus levofloxacin at 250 milligrams, with an option to switch to oral therapy, for treatment of complicated lower urinary tract infection and pyelonephritis. Antimicrobial agents and chemotherapy. 2009 Sep 1;53(9):3782-92.

154. Breedt J, Teras J, Gardovskis J, Maritz FJ, Vaasna T, Ross DP, Gioud-Paquet M, Dartois N, Ellis-Grosse EJ, Loh E. Safety and efficacy of tigecycline in treatment of skin and skin structure infections: results of a double-blind phase 3 comparison study with vancomycin-aztreonam. Antimicrobial agents and chemotherapy. 2005 Nov 1;49(11):4658-66.

Appendix Table 3. Comparison of ITT to PP CI using Newcombe method

|  | PP  Median (IQR) | ITT  Median (IQR) | Difference  ITT - PP  Median  (IQR) | Wilcoxon signed-rank test p-value | PP analysis is more conservative  N (%) |
| --- | --- | --- | --- | --- | --- |
| CI width | 12.4  (9.7, 16.6) | 13.4  (11.4, 17.5) | 1.0  (-0.3, 2.1) | <0.0001 | 52 (31.7%) |
| Lower CI limit | -6.9  (-10.0, -4.0) | -7.5  (-10.2, -4.8) | -0.5  (-2.0, 1.1) | 0.0249 | 69 (42.1%) |

CI = confidence interval; IQR = interquartile range; ITT = intention-to-treat; PP = per-protocol

A positive value for the difference in CI width indicates less precise estimation of the ARR with ITT analysis. A negative difference for the lower CI limit signifies that the PP lower CI limit lies above the ITT CI limit, so ITT analysis has a more conservative finding.

Appendix Table 4. Agreement between ITT and PP analyses for conclusion on non-inferiority

|  | Non-inferiority proven in ITT analysis | Inconclusive in ITT analysis |
| --- | --- | --- |
| Non-inferiority proven in PP analysis | 121 | 12 |
| Inconclusive in PP analysis | 7 | 22 |

Two comparisons did not provide a non-inferiority margin

Appendix Table 5. Multivariable linear regression of difference between ITT lower CI and PP lower CI weighted by sample size in ITT population

| Predictors | Co-efficient (95% CI) | P-value |
| --- | --- | --- |
| PP exclusion of concomitant therapy | -0.42 (-1.45 to 0.61) | 0.4181 |
| Low risk for allocation concealment bias | -0.49 (-1.43 to 0.46) | 0.3086 |
| Low risk for performance bias | -1.22 (-2.68 to 0.23) | 0.0985 |
| Low risk for detection bias | 1.08 (-0.50 to 2.67) | 0.1791 |
| Proportion of treatment arm in the ITT population that was included in the PP population per every 10% | 3.59 (2.81 to 4.36) | <0.0001 |
| Proportion of control arm in the ITT population that was included in the PP population per every 10% | -3.25 (-3.98 to -2.52) | <0.0001 |

CI = confidence interval; ITT = intention-to-treat; PP = per-protocol

The weights were equal to the sample size of the ITT population in each study.

The dependent variable in the model is ITT lower CI limit minus PP lower CI limit. Therefore, a negative co-efficient is associated with a smaller ITT lower CI limit, so the ITT analysis is more conservative than PP analysis. Conversely, a positive co-efficient is associated with a smaller PP lower CI limit, so the PP analysis is more conservative than the ITT analysis.

**Appendix Figure 1. Forest plot of point estimate and CI for ITT and PP analyses**

CI = confidence interval; ITT = intention to treat; PP = per protocol

**Appendix Figure 2. Point estimate and lower CI limit difference between ITT and PP analyses**

CI = confidence interval; ITT = intention to treat; PP = per protocol

**Appendix Figure 3. ARR of excluded versus per-protocol population**

ARR = absolute risk reduction in %; PP = per-protocol

A diagonal line is drawn at y=x, where points below the line indicates cases where ARR in excluded population is worse than the ARR in the PP population

**Appendix Figure 4. Success rate of treatment and control arms combined for excluded versus per-protocol population**

PP = per-protocol

Line has equation of y=x, so points below the line signifies that the success rate in the excluded population is lower than the PP population

**Appendix Figure 5. Success rate of treatment arm for excluded versus per-protocol population**

PP = per-protocol

Line has equation of y=x, so points below the line signifies that the success rate in the excluded population is lower than the PP population

**Appendix Figure 6. Success rate of control arm for excluded versus per-protocol population**

PP = per-protocol

Line has equation of y=x, so points below the line signifies that the success rate in the excluded population is lower than the PP population

**Appendix Figure 7. Funnel plot of both ITT and PP analyses**

ITT = intention-to-treat; PP = per-protocol

The Egger’s regression test for funnel plot asymmetry had a p-value of 0.9132.

**Appendix Figure 8. Funnel plot for only ITT analyses including excluded studies**

Blue = studies included in the analysis that reported both ITT and PP analyses; Red = studies excluded from analysis because they reported ITT analysis and did not report PP analysis; ITT = intention-to-treat; PP = per-protocol

The Egger’s regression test for funnel plot asymmetry had a p-value of 0.4373.

**Appendix Figure 9. Funnel plot for only PP analyses including excluded studies**

Blue = studies included in the analysis that reported both ITT and PP analyses; Red = studies excluded from analysis because they reported PP analysis and did not report ITT analysis; ITT = intention-to-treat; PP = per-protocol

The Egger’s regression test for funnel plot asymmetry had a p-value of 0.5556.

**Appendix Text 1. PRISMA Checklist**

| Section / topic | # | Checklist item | Reported on page # |
| --- | --- | --- | --- |
| TITLE |  |  |  |
| Title | 1 | Identify the report as a systematic review, meta-analysis, or both. | 1 |
| ABSTRACT |  |  |  |
| Structured summary | 2 | Provide a structured summary including, as applicable: background; objectives; data sources; study eligibility criteria, participants, and interventions; study appraisal and synthesis methods; results; limitations; conclusions and implications of key findings; systematic review registration number. | 3, 4 |
| INTRODUCTION |  |  |  |
| Rationale | 3 | Describe the rationale for the review in the context of what is already known. | 5, 6 |
| Objectives | 4 | Provide an explicit statement of questions being addressed with reference to participants, interventions, comparisons, outcomes, and study design (PICOS). | 6 |
| METHODS |  |  |  |
| Protocol and registration | 5 | Indicate if a review protocol exists, if and where it can be accessed (e.g., Web address), and, if available, provide registration information including registration number. | 7 |
| Eligibility criteria | 6 | Specify study characteristics (e.g., PICOS, length of follow-up) and report characteristics (e.g., years considered, language, publication status) used as criteria for eligibility, giving rationale. | 7, 8 |
| Information sources | 7 | Describe all information sources (e.g., databases with dates of coverage, contact with study authors to identify additional studies) in the search and date last searched. | 7 |
| Search | 8 | Present full electronic search strategy for at least one database, including any limits used, such that it could be repeated. | Appendix Text 2 |
| Study selection | 9 | State the process for selecting studies (i.e., screening, eligibility, included in systematic review, and, if applicable, included in the meta-analysis). | 7, 8 |
| Data collection process | 10 | Describe method of data extraction from reports (e.g., piloted forms, independently, in duplicate) and any processes for obtaining and confirming data from investigators. | 8 |
| Data items | 11 | List and define all variables for which data were sought (e.g., PICOS, funding sources) and any assumptions and simplifications made. | 8 |
| Risk of bias in individual studies | 12 | Describe methods used for assessing risk of bias of individual studies (including specification of whether this was done at the study or outcome level), and how this information is to be used in any data synthesis. | 10 |
| Summary measures | 13 | State the principal summary measures (e.g., risk ratio, difference in means). | 8, 9 |
| Synthesis of results | 14 | Describe the methods of handling data and combining results of studies, if done, including measures of consistency (e.g., I^2^) for each meta-analysis. | 9 - 11 |
| Risk of bias across studies | 15 | Specify any assessment of risk of bias that may affect the cumulative evidence (e.g., publication bias, selective reporting within studies). | 9, 10 |
| Additional analyses | 16 | Describe methods of additional analyses (e.g., sensitivity or subgroup analyses, meta-regression), if done, indicating which were pre-specified. | 10, 11 |
| RESULTS |  |  |  |
| Study selection | 17 | Give numbers of studies screened, assessed for eligibility, and included in the review, with reasons for exclusions at each stage, ideally with a flow diagram. | Figure 2 |
| Study characteristics | 18 | For each study, present characteristics for which data were extracted (e.g., study size, PICOS, follow-up period) and provide the citations. | Appendix Table 1 |
| Risk of bias within studies | 19 | Present data on risk of bias of each study and, if available, any outcome level assessment (see item 12). | Table 2  Appendix Table 2 |
| Results of individual studies | 20 | For all outcomes considered (benefits or harms), present, for each study: (a) simple summary data for each intervention group (b) effect estimates and confidence intervals, ideally with a forest plot. | Appendix Table 1  Appendix Figure 1 |
| Synthesis of results | 21 | Present results of each meta-analysis done, including confidence intervals and measures of consistency. | N/A |
| Risk of bias across studies | 22 | Present results of any assessment of risk of bias across studies (see Item 15). | Appendix Figures 7 – 9 |
| Additional analysis | 23 | Give results of additional analyses, if done (e.g., sensitivity or subgroup analyses, meta-regression [see Item 16]). | Table 4, 5 |
| DISCUSSION |  |  |  |
| Summary of evidence | 24 | Summarize the main findings including the strength of evidence for each main outcome; consider their relevance to key groups (e.g., healthcare providers, users, and policy makers). | 15 |
| Limitations | 25 | Discuss limitations at study and outcome level (e.g., risk of bias), and at review-level (e.g., incomplete retrieval of identified research, reporting bias). | 18, 19 |
| Conclusions | 26 | Provide a general interpretation of the results in the context of other evidence, and implications for future research. | 15 - 18 |
| FUNDING |  |  |  |
| Funding | 27 | Describe sources of funding for the systematic review and other support (e.g., supply of data); role of funders for the systematic review. | 20 |

**Appendix Text 2. Literature search strategy**

Database: Ovid MEDLINE(R) and Epub Ahead of Print, In-Process & Other Non-

Indexed Citations, Daily and Versions(R) <1946 to November 22, 2019>

Search Strategy:

--------------------------------------------------------------------------------

1 non-inferiority trial.mp. or exp Equivalence Trial/ (1417)

2 noninferior*.mp. (5296)

3 non-inferior*.mp. (6292)

4 1 or 2 or 3 (11628)

5 exp Anti-Bacterial Agents/ (710985)

6 antibiotic*.mp. (365238)

7 beta lactam*/ or penicillin*/ or cephalosporin*/ or carbapenem*/ or

aminoglycoside*/ or glycopeptide*/ or fluoroqunolone*/ or quinolone*/ or

oxazolidinone*/ or tetracycline*/ or macrolide*/ or ansamycin*/ or

streptogramin*/ or lipopeptide*/ or sulfonamide*/ or monobactam*/ or

nitrofuran*/ or lincosamide*/ or polypeptide*.mp. [mp=title, abstract, original title,

name of substance word, subject heading word, floating sub-heading word, keyword

heading word, organism supplementary concept word, protocol supplementary

concept word, rare disease supplementary concept word, unique identifier,

synonyms] (125516)

8 amikacin*/ or gentamicin*/ or kanamycin*/ or neomycin*/ or netilmicin*/ or

tobramycin*/ or paromomycin*/ or streptomycin*/ or spectinomycin*/ or

rifaximin*/ or ertapenem*/ or doripenem*/ or imipenem*/ or meropenem*/ or

cefadroxil*/ or cefazolin*/ or cephradine*/ or cephapirin*/ or cephalothin*/ or

cefalexin*/ or cephalexin*/ or cefaclor*/ or cefoxitin*/ or cefotetan*/ or

cefamandole*/ or cefmetazole*/ or cefonicid*/ or loracarbef*/ or cefprozil*/ or

cefuroxime*/ or cefixime*/ or cefdinir*/ or cefditoren*/ or cefoperazone*/ or

cefotaxime*/ or cefpodoxime*/ or ceftazidime*/ or ceftibuten*/ or ceftizoxime*/ or

moxalactam*/ or ceftriaxone*/ or cefepime*/ or ceftaroline*/ or ceftobiprole*/ or

teicoplanin*/ or vancomycin*/ or telavancin*/ or dalbavancin*/ or oritavancin*/ or

clindamycin*/ or lincomycin*/ or daptomycin*/ or azithromycin*/ or

clarithromycin*/ or erythromycin*/ or roxithromycin*/ or telithromycin*/ or

spiramycin*/ or fidaxomicin*/ or aztreonam*/ or furazolidone*/ or

nitrofurantoin*/ or linezolid*/ or posizolid*/ or radezolid*/ or torezolid*/ or

amoxicillin*/ or ampicillin*/ or azlocillin*/ or dicloxacillin*/ or flucloxacillin*/ or

cloxacillin*/ or mezlocillin*/ or methicillin*/ or nafcillin*/ or oxacillin*/ or

piperacillin*/ or temocillin*/ or ticarcillin*/ or bacitracin*/ or colistin*/ or

polymyxin*/ or ciprofloxacin*/ or enoxacin*/ or gatifloxacin*/ or gemifloxacin*/ or

levofloxacin*/ or lomefloxacin*/ or moxifloxacin*/ or nadifloxacin*/ or nalidixic*/

or norfloxacin*/ or ofloxacin*/ or trovafloxacin*/ or grepafloxacin*/ or

sparfloxacin*/ or temafloxacin*/ or mafenide*/ or sulfacetamide*/ or sulfadiazine*/

or sulfadimethoxine*/ or sulfamethizole*/ or sulfamethoxazole*/ or sulfanilimide*/

or sulfasalazine*/ or sulfisoxazole*/ or Septra/ or trimethoprim*/ or

sulfonamidochrysoidine*/ or demeclocycline*/ or doxycycline*/ or metacycline*/

or minocycline*/ or oxytetracycline*/ or clofazimine*/ or dapsone*/ or

capreomycin*/ or cycloserine*/ or ethambutol*/ or ethionamide*/ or isoniazid*/ or

pyrazinamide*/ or rifampicin*/ or rifampin*/ or rifabutin*/ or rifapentine*/ or

streptomycin*/ or arsphenamine*/ or chloramphenicol*/ or fosfomycin*/ or fusidic

acid*/ or metronidazole*/ or mupirocin*/ or platensimycin*/ or quinupristin*/ or

thiamphenicol*/ or tigecycline*/ or tinidazole*.mp. [mp=title, abstract, original title,

name of substance word, subject heading word, floating sub-heading word, keyword

heading word, organism supplementary concept word, protocol supplementary

concept word, rare disease supplementary concept word, unique identifier,

synonyms] (8202)

9 5 or 6 or 7 or 8 (1001463)

10 4 and 9 (1202)

11 ((randomized controlled trial or controlled clinical trial).pt. or randomized.ab. or

placebo.ab. or clinical trials as topic.sh. or randomly.ab. or trial.ti. or clinical trial.mp.

or clinical trial.pt. or random*.mp.) not (animals not (humans and animals)).sh.

(1677664)

12 10 and 11 (940)

Database: Embase <1974 to 2019 November 22>

Search Strategy:

--------------------------------------------------------------------------------

1 non-inferiority trial.mp. or exp Equivalence Trial/ (2205)

2 noninferior*.mp. (7437)

3 non-inferior*.mp. (13278)

4 1 or 2 or 3 (19853)

5 exp Anti-Bacterial Agents/ (3341534)

6 antibiotic*.mp. (727134)

7 beta lactam*/ or penicillin*/ or cephalosporin*/ or carbapenem*/ or

aminoglycoside*/ or glycopeptide*/ or fluoroqunolone*/ or quinolone*/ or

oxazolidinone*/ or tetracycline*/ or macrolide*/ or ansamycin*/ or

streptogramin*/ or lipopeptide*/ or sulfonamide*/ or monobactam*/ or

nitrofuran*/ or lincosamide*/ or polypeptide*.mp. [mp=title, abstract, heading

word, drug trade name, original title, device manufacturer, drug manufacturer,

device trade name, keyword, floating subheading word, candidate term word]

(163892)

8 amikacin*/ or gentamicin*/ or kanamycin*/ or neomycin*/ or netilmicin*/ or

tobramycin*/ or paromomycin*/ or streptomycin*/ or spectinomycin*/ or

rifaximin*/ or ertapenem*/ or doripenem*/ or imipenem*/ or meropenem*/ or

cefadroxil*/ or cefazolin*/ or cephradine*/ or cephapirin*/ or cephalothin*/ or

cefalexin*/ or cephalexin*/ or cefaclor*/ or cefoxitin*/ or cefotetan*/ or

cefamandole*/ or cefmetazole*/ or cefonicid*/ or loracarbef*/ or cefprozil*/ or

cefuroxime*/ or cefixime*/ or cefdinir*/ or cefditoren*/ or cefoperazone*/ or

cefotaxime*/ or cefpodoxime*/ or ceftazidime*/ or ceftibuten*/ or ceftizoxime*/ or

moxalactam*/ or ceftriaxone*/ or cefepime*/ or ceftaroline*/ or ceftobiprole*/ or

teicoplanin*/ or vancomycin*/ or telavancin*/ or dalbavancin*/ or oritavancin*/ or

clindamycin*/ or lincomycin*/ or daptomycin*/ or azithromycin*/ or

clarithromycin*/ or erythromycin*/ or roxithromycin*/ or telithromycin*/ or

spiramycin*/ or fidaxomicin*/ or aztreonam*/ or furazolidone*/ or

nitrofurantoin*/ or linezolid*/ or posizolid*/ or radezolid*/ or torezolid*/ or

amoxicillin*/ or ampicillin*/ or azlocillin*/ or dicloxacillin*/ or flucloxacillin*/ or

cloxacillin*/ or mezlocillin*/ or methicillin*/ or nafcillin*/ or oxacillin*/ or

piperacillin*/ or temocillin*/ or ticarcillin*/ or bacitracin*/ or colistin*/ or

polymyxin*/ or ciprofloxacin*/ or enoxacin*/ or gatifloxacin*/ or gemifloxacin*/ or

levofloxacin*/ or lomefloxacin*/ or moxifloxacin*/ or nadifloxacin*/ or nalidixic*/

or norfloxacin*/ or ofloxacin*/ or trovafloxacin*/ or grepafloxacin*/ or

sparfloxacin*/ or temafloxacin*/ or mafenide*/ or sulfacetamide*/ or sulfadiazine*/

or sulfadimethoxine*/ or sulfamethizole*/ or sulfamethoxazole*/ or sulfanilimide*/

or sulfasalazine*/ or sulfisoxazole*/ or Septra/ or trimethoprim*/ or

sulfonamidochrysoidine*/ or demeclocycline*/ or doxycycline*/ or metacycline*/

or minocycline*/ or oxytetracycline*/ or clofazimine*/ or dapsone*/ or

capreomycin*/ or cycloserine*/ or ethambutol*/ or ethionamide*/ or isoniazid*/ or

pyrazinamide*/ or rifampicin*/ or rifampin*/ or rifabutin*/ or rifapentine*/ or

streptomycin*/ or arsphenamine*/ or chloramphenicol*/ or fosfomycin*/ or fusidic

acid*/ or metronidazole*/ or mupirocin*/ or platensimycin*/ or quinupristin*/ or

thiamphenicol*/ or tigecycline*/ or tinidazole*.mp. [mp=title, abstract, heading

word, drug trade name, original title, device manufacturer, drug manufacturer,

device trade name, keyword, floating subheading word, candidate term word]

(82434)

9 5 or 6 or 7 or 8 (3589851)

10 4 and 9 (4140)

11 (placebo.sh. or controlled study.ab. or random*.ti,ab. or trial*.ti,ab. or clinical

trial.mp. or clinical trial.pt. or random*.mp.) not (animals not (humans and

animals)).sh. (3122450)

12 10 and 11 (3756)

Cochrane CENTRAL

non-inferiority trial OR non-inferior* OR noninferior*

AND

antibacterial agents OR antibiotic* OR beta lactam* OR penicillin* OR

cephalosporin* OR carbapenem* OR aminoglycoside* OR glycopeptide* OR

fluoroqunolone* OR quinolone* OR oxazolidinone* OR tetracycline* OR macrolide*

OR ansamycin* OR streptogramin* OR lipopeptide* OR sulfonamide* OR

monobactam* OR nitrofuran* OR lincosamide* OR polypeptide*

OR amikacin* OR gentamicin* OR kanamycin* OR neomycin* OR netilmicin* OR

tobramycin* OR paromomycin* OR streptomycin* OR spectinomycin* OR rifaximin*

OR ertapenem* OR doripenem* OR imipenem* OR meropenem* OR cefadroxil* OR

cefazolin* OR cephradine* OR cephapirin* OR cephalothin* OR cefalexin* OR

cephalexin* OR cefaclor* OR cefoxitin* OR cefotetan* OR cefamandole* OR

cefmetazole* OR cefonicid* OR loracarbef* OR cefprozil* OR cefuroxime* OR

cefixime* OR cefdinir* OR cefditoren* OR cefoperazone* OR cefotaxime* OR

cefpodoxime* OR ceftazidime* OR ceftibuten* OR ceftizoxime* OR moxalactam* OR

ceftriaxone* OR cefepime* OR ceftaroline* OR ceftobiprole* OR teicoplanin* OR

vancomycin* OR telavancin* OR dalbavancin* OR oritavancin* OR clindamycin* OR

lincomycin* OR daptomycin* OR azithromycin* OR clarithromycin* OR

erythromycin* OR roxithromycin* OR telithromycin* OR spiramycin* OR

fidaxomicin* OR aztreonam* OR furazolidone* OR nitrofurantoin* OR linezolid* OR

posizolid* OR radezolid* OR torezolid* OR amoxicillin* OR ampicillin* OR azlocillin*

OR dicloxacillin* OR flucloxacillin* OR cloxacillin* OR mezlocillin* OR methicillin*

OR nafcillin* OR oxacillin* OR piperacillin* OR temocillin* OR ticarcillin* OR

bacitracin* OR colistin* OR polymyxin* OR ciprofloxacin* OR enoxacin* OR

gatifloxacin* OR gemifloxacin* OR levofloxacin* OR lomefloxacin* OR moxifloxacin*

OR nadifloxacin* OR nalidixic* OR norfloxacin* OR ofloxacin* OR trovafloxacin* OR

grepafloxacin* OR sparfloxacin* OR temafloxacin* OR mafenide* OR sulfacetamide*

OR sulfadiazine* OR sulfadimethoxine* OR sulfamethizole* OR sulfamethoxazole*

OR sulfanilimide* OR sulfasalazine* OR sulfisoxazole* OR Septra OR trimethoprim*

OR sulfonamidochrysoidine* OR demeclocycline* OR doxycycline* OR metacycline*

OR minocycline* OR oxytetracycline* OR clofazimine* OR dapsone* OR

capreomycin* OR cycloserine* OR ethambutol* OR ethionamide* OR isoniazid* OR

pyrazinamide* OR rifampicin* OR rifampin* OR rifabutin* OR rifapentine* OR

streptomycin* OR arsphenamine* OR chloramphenicol* OR fosfomycin* OR fusidic

acid* OR metronidazole* OR mupirocin* OR platensimycin* OR quinupristin* OR

thiamphenicol* OR tigecycline* OR tinidazole*
